# Chronic conditions and healthcare cost and utilization among underserved Medicare beneficiaries

**DOI:** 10.1371/journal.pone.0340785

**Published:** 2026-02-26

**Authors:** Karen Fredriksen Goldsen, Hyun-Jun Kim, Natalie R. Turner, Charles A. Emlet

**Affiliations:** 1 Goldsen Institute, School of Social Work, University of Washington, Seattle, Washington, United States of America; 2 School of Social Work & Criminal Justice, University of Washington, Tacoma, Washington, United States of America; University of New South Wales, AUSTRALIA

## Abstract

Underserved older adults face increased risk for certain chronic conditions and multimorbidity, yet research on healthcare spending and utilization in these groups is limited. For example, there is a glaring absence of research on sexual and gender minority (SGM) Medicare beneficiaries. To address this gap, this study uses linked data from Aging with Pride: National Health, Aging and Sexuality/Gender Study (NHAS) and CMS Chronic Conditions Warehouse data (n = 902) to examine chronic conditions, healthcare spending and utilization among a diverse sample of SGM older adult Medicare beneficiaries. Chronic condition complexity was identified using the Medicare Chronic Conditions/Other Chronic Conditions files. Additional explanatory variables included adverse experiences, psychological and social resources, health-related indicators, socioeconomic factors, and background characteristics. The Cost and Use file was used to calculate four outcome variables: total Medicare spending, spending on physician services, high-cost beneficiary status, and healthcare utilization. A series of linear and logistic regressions were used to estimate the association between explanatory and outcome variables. SGM older adult participants with the greatest severity and complexity of chronic conditions had significantly higher total Medicare spending, spending on physician services, and were more likely to be a high-cost beneficiary and higher use of healthcare services compared to those who were comparatively healthy. We also find strong evidence linking higher Medicare spending to disability and dual eligibility, highlighting an urgent need for research given SGM older adults’ increased risk for disabling chronic conditions, yet at times lower healthcare utilization. Higher day-to-day discrimination was associated with greater likelihood of chronic condition complexity and lower Medicare spending. Understanding the relationship between chronic health conditions and healthcare cost and utilization is a critical step in developing responsive health services and effective interventions to promote healthy aging in our increasingly diverse yet often underserved communities.

## Introduction

The population of older adults in the United States has been rapidly increasing and becoming more diverse [1]. Yet many diverse older adults remain underserved and are at greater risk for some chronic conditions and multimorbidity. Among underserved populations there remains a dearth of research on healthcare spending and utilization, including among the growing population of sexual and gender minority (SGM) older adults. One estimation showed by 2060, the number of adults age 50 and older who self-identified as SGM would be over five million [2]. There has been increasing recognition in the importance of understanding and ameliorating health disparities among SGM older adults. Both NIH [3] and Healthy People 2030 [4] have highlighted the need for increased attention and efforts to address health disparities among SGM.

A primary driver of healthcare spending and utilization among older adults is the burden of disability and compromised physical and mental health. Existing research has documented extensive health disparities among SGM older adults, specifically in relation to chronic conditions and multi-morbidity. National population studies have shown that lesbian, gay, and bisexual older adults were more likely to report having nine out of 12 chronic conditions compared to heterosexuals of similar age, and they were more likely to report higher rates of disability, mental distress, weakened immune systems and low back or neck pain [5]. Other studies have found lesbian, gay, and bisexual older adults were more likely to report having multiple physical and chronic conditions than their heterosexual counterparts [5–8]. When examining the association between gender identity and health, transgender older adults were at significantly higher risk for poor physical health, disability, and depressive symptomology compared to their non-transgender counterparts [9,10].

There are additional important differences by subgroups. Sexual minority older women were more likely to report a higher number of chronic conditions as well as more incidence of arthritis, asthma, cardiovascular disease, stroke, and obesity compared to heterosexual older women [5,11]. Sexual minority older men were more likely to report having angina pectoris, cancer, hypertension and diabetes compared to their heterosexual counterparts [5]. Using nationally representative datasets of US adults, Dyar et al. [7] and Fredriksen-Goldsen et al. [5] found that bisexual adults had a higher risk for some physical health conditions and adverse health indicators compared with other sexual minority groups. In terms of gender identity, Pharr [12], using the 2019 Behavioral Risk Fact Surveillance System survey with 31 states found that nonbinary older adults had the highest rates of disability compared with other SGM subgroups, including transgender men and women. Guo et al. [13] found that in Florida, transgender adults had higher prevalence of specific chronic conditions compared to cisgender adults.

Existing research among older adults in general has found several factors associated with healthcare spending and utilization. Overall, older adults 65 and over accounted for higher spending [14] and use of healthcare [15,16] compared to individuals under age 65. Disability and lower physical functioning were also associated with increased healthcare spending and utilization among older adults [17,18]. Dual eligibility status (being dually eligible for Medicare and Medicaid) was associated with increased healthcare spending and utilization [19,20]. Regarding socioeconomic factors, older adults living below the poverty level were more likely to have multiple chronic conditions and yet were less likely to use healthcare services compared to wealthier individuals [21,22]. Lower income has been strongly associated with increased barriers to accessing care, potentially resulting in lower healthcare utilization [21]. Higher educational attainment was associated with lower probability of hospitalizations among middle-age and older adults [23], while older women had fewer physician visits and hospitalizations compared to older men with similar health needs [24]. Older adults of color had lower healthcare utilization than White older adults [25].

Behavioral health was also associated with healthcare spending and utilization. For example, smoking was associated with increased healthcare spending among older adults [26]. Older adults who reported binge drinking had fewer physician visits, reduced preventive care, and increased ED visits [27–29] compared to those who did not report binge drinking. Increased alcohol consumption among older adults was consistent with increased acute-care utilization [28].

In terms of social factors, living alone was associated with greater use of primary care services, number of hospital admissions and higher inpatient spending among older adults [30,31]. Marriage was associated with reduced use of inpatient services and skilled nursing facilities and increased use of outpatient services [32]. Loneliness was positively associated with physician visits among older adults, but not with hospitalizations [33]. One systematic review of the relationship between social relationships and healthcare among older adults found that weaker social relationships overall were associated with increased rates of hospital readmissions [34], but were not linked to ambulatory healthcare services, such as physician visits and home-based services. Gao et al. [35] found that older adults with greater social, cultural, and community engagement reported lower inpatient service utilization and higher outpatient service utilization, indicating that such engagement may have protective effects in shaping early and preventive care.

There has been limited prior research on healthcare spending and utilization among SGM populations. One study examined healthcare spending between same-sex couples using the Medical Expenditure Panel Survey (MEPS). Men in same-sex couples experienced higher spending on prescription drugs, specifically antiretroviral medications and psychotropic medications. There were no differences found in healthcare spending between women in same-sex and different-sex couples [36].

The research that has been conducted on healthcare utilization among SGM populations suggests that lesbian, gay, and bisexual individuals of all ages report lower use of routine checkups, inpatient, and emergency health services compared to heterosexual counterparts [37–39]. Among older adults, gender minority older adults were more likely to delay or not receive care due to cost compared to heterosexual older adults [12]. Additionally, older nonbinary adults, transgender women, and bisexual men were less likely to have a regular healthcare provider compared to other SGM subgroups [12]. These patterns of underutilization, combined with a higher risk of chronic conditions and disability, create a complex and poorly understood picture of healthcare engagement among SGM older adults and warrant further investigation of their real-world service use and costs using rigorous data.

To date, prior research on SGM health disparities has relied heavily on self-report, which poses limitations. Relying only on self-report, for example, over a long period of time, may result in under-reporting visits to a healthcare provider [40]. Increasingly, administrative health data has been shown to be especially useful for health services research, particularly for chronic diseases that require ongoing contact with the healthcare system [41,42]. Existing research found that administrative data were much more reliable than self-report in examining chronic condition diagnoses over one’s lifetime and healthcare spending and utilization accrued over a one-year period. For example, in the short-term (about 30 days), self-report and administrative data were equivalent, but when asked to recall diagnoses or healthcare visits beyond that timeframe, patient accuracy became questionable [40,41]. Additionally, different diagnoses or healthcare events ranged in difficulty for individuals to recall. For example, diagnoses that were less familiar to individuals with intermittent and nonspecific symptoms (e.g., heart failure) were underreported via self-reports. Conversely, diseases that were well-known by individuals (e.g., stroke) were overreported in self-reports, as individuals attribute symptoms to the disease [41].

CMS administrative health data was found to be particularly valuable for health services research, with 98% of adults ages 65 and over estimated to be enrolled in Medicare, thus, making Medicare data an incredibly rich source of information on healthcare spending and utilization for the older adult population [43]. The information on health conditions and healthcare spending and utilization has been largely considered reliable and valid [43]. Using CMS health records information also allowed for larger sample sizes and reduced challenges with attrition, non-response, and measurement error [44,45]. Its structure allowed researchers to follow individuals over a long period of time to examine the long-term effects of health conditions on healthcare spending and utilization among beneficiaries. Given Medicare’s reach among those over the age of 65, CMS administrative health data can provide insights into health disparities at a large, population-level scale [43]. However, to date little is known about SGM older adults’ health disparities based on administrative health records as well as healthcare spending and utilization.

To date there has been limited use of administrative health data in understanding SGM health disparities, healthcare spending and cost. A few existing studies have reported identifying transgender Medicare beneficiaries by using a diagnosis-code algorithm [46,47] which identified individuals with diagnosis codes for gender identity disorders (e.g., ‘transsexualism,’ ‘gender identity disorder in children or adults’). Using this approach, transgender Medicare beneficiaries were found to have more chronic conditions, and were more likely to be diagnosed with asthma, autism spectrum disorder, chronic obstructive pulmonary disease (COPD), HIV, hepatitis, depression, schizophrenia, and substance use disorder compared with cisgender beneficiaries [46]. Transgender Medicare beneficiaries were also shown to have higher rates of emergency department visits, hospitalizations, and mental health care use compared to cisgender beneficiaries [47,48].

While accessing administrative health data on health conditions and healthcare spending and utilization is an important step forward for understanding health disparities among transgender beneficiaries, this approach for identifying transgender beneficiaries has significant limitations. For example, using diagnostic codes to identify transgender beneficiaries may have misidentified transgender beneficiaries as cisgender if their identity or transition status was not reflected in specific disease classification codes used by providers, in this case International Classification of Diseases (ICD)-9/ICD-10 codes. Additionally, approaches to identifying sexual minority individuals that only examine sexual minorities in same-sex partnerships exclude those that are not married, single, divorced, or those that are a sexual minority in an opposite-sex relationship.

The present study addresses these critical methodological gaps by linking survey data from the Aging with Pride: National Health, Aging and Sexuality/Gender Study (NHAS) with administrative health data. This linkage provides a unique opportunity to examine health conditions and healthcare spending and utilization among a demographically diverse sample of SGM older adults and allows for investigating specific modifiable factors that may contribute to chronic condition complexity as well as to what extent disparities in chronic conditions are linked to both healthcare utilization and healthcare spending. To understand health disparities and to identify underlying mechanisms and modifiable factors that account for healthcare spending and utilization among SGM older adults, we utilized the Health Equity Promotion Model (HEPM). The HEPM is a framework oriented toward SGM people reaching their full mental and physical health potential that considers both adverse and positive health-related circumstances. The model highlights (a) social location, heterogeneity and intersectionality within SGM communities; (b) the influence of structural and environmental context; and (c) both health-promoting and adverse pathways that encompass behavioral, social, psychological, and biological processes [49]. The HEPM also expands upon earlier conceptualizations of SGM health by integrating a life course development perspective within the health-promotion model. By integrating service utilization as well as the deleterious effect of barriers to care and non-utilization on health outcomes, this study utilized an approach similar to Joynt et al. [50] and Rivera-Hernandez et al. [51], who identified high-cost beneficiaries and classified Medicare beneficiaries on the basis of multimorbidity and chronic condition complexity. In this paper, we addressed the following research questions:

1. How does the severity of chronic health conditions among SGM older adults differ by background characteristics/social location, key health indicators, and social determinants of health, including adverse experiences, psychological and social resources, and socioeconomic factors?
2. To what extent do healthcare spending and utilization vary by the severity of chronic conditions/multimorbidity among SGM older adults?
3. Do background characteristics/social location, key health indicators, and social determinants of health, including adverse experiences, psychological and social resources, and socioeconomic factors in addition to the severity of chronic conditions, predict healthcare spending and utilization?
4. What accounts for Medicare spending: Is it age or reason for Medicare entitlement (disability)?

## Methods

### Data

To address these research questions, we linked data from the 2014 NHAS [2] and the following 2014 CMS Chronic Conditions Warehouse data files: Medicare Beneficiary Summary File (MBSF), Cost and Use, Chronic Conditions and Other Chronic Conditions. NHAS is the first national and federally funded longitudinal study to examine the health and well-being of SGM older adults, and this study utilized the baseline data collected from October 2014 to August 2015. The survey cover letter provided the elements of consent and return of the completed survey implied consent from the participant. Inclusion criteria included being age 50 and older and self-identification as SGM. A total of 2,450 participants completed the questionnaire. In 2020, 2,217 active NHAS participants were contacted and asked to voluntarily give permission to link their survey data with CMS data and provide their Medicare Beneficiary Identifier (MBI) and/or social security number (SSN), and 1,465 granted permission for the linkage with 640 providing their MBI and 350 providing their SSN. This study was approved by the University of Washington IRB. A waiver for informed consent was granted by the University of Washington IRB based on the common rule [45 CFR § 46.116(d)]. A Data Use Agreement was established between CMS and NHAS. The Medicare Research Information Center, with support from the National Institute on Aging, identified CMS data matched with eligible NHAS participants based on MBI, SSN, date of birth, first and last names, and zip code. We obtained the 2014 CMS data in March 2022, and we successfully linked the 2014 NHAS and CMS data after obtaining IRB approval of modification in 2024 for a total of 902 participants. Participant IDs and names were stored separately and only the ID was linked to the study data.

Measures used in this study are detailed in Table 1. The outcome variables of interest were total Medicare spending, spending on physician services, high-cost beneficiary status and healthcare utilization. Total Medicare spending and spending on physician services were each calculated per beneficiary as the sum of Medicare, primary payer, co-insurance and deductible payments. Total Medicare spending included CMS Cost and Use claims for inpatient, outpatient, physician, post-acute, and other services. See Supplementary S1 Table for a complete list of included services from the Cost and Use file. Separate analyses for other services, including hospitalizations and post-acute care, were not examined due to high numbers of participants with zero spending in these categories (95.01% and 97.45%, respectively). This method of measuring total Medicare spending has been used previously [51]. High-cost beneficiary status was dichotomized and defined as beneficiaries in the highest 10% of total Medicare spending among all beneficiaries (in top 10% of total spending vs. not). Healthcare utilization was dichotomized (no use vs. any use) and included the same CMS Cost and Use claims services used in Total Medicare spending.

**Table 1:** Description of measures.

Variables	Descriptions
Outcome variables	
Total Medicare spending	We measured total Medicare spending by taking the sum of allowed payments by Medicare, primary payer, co-insurance and deductible payments in the year based on cost and utilization claims. For total Medicare spending, inpatient, outpatient, emergency, physician, and post-acute services were summed.
Total spending on physician services	For physician services, Part B Physician Services and Evaluation and Management costs were summed [51,52]
High-cost beneficiaries	We categorized high-cost beneficiaries using a dichotomized summary variable (highest 10% of total Medicare spending vs. not in highest 10% of total Medicare spending) [50,51,53]
Healthcare utilization	We measured healthcare utilization with a dichotomized summary variable (no use vs. any use) to characterize health services utilization for the year. Participants were coded for any use if they used any of the following in 2014: skilled nursing facility, home health, hospitalization (inpatient and outpatient), or ER [51]
Explanatory Variables	
Chronic conditions groups	Participants are categorized into one of four chronic conditions groups using diagnostic information from CMS Chronic Conditions and Other Chronic Conditions data files. 1) Major complex chronic illness includes participants who have ever been diagnosed with at least three complex chronic conditions or at least six other chronic conditions; 2) Minor complex chronic illness includes participants who have ever been diagnosed with one or two complex chronic conditions and less than six other chronic conditions; 3) Simple chronic illness includes participant who have never been diagnosed with any complex chronic conditions and have been diagnosed with one to five other chronic conditions; 4) Comparatively healthy includes participants who have no chronic conditions [50,51]
Adverse experiences	*Lifetime victimization* was measured using a summed score (range: 0–27) of how often individuals experienced nine types of victimization (e.g., threatened with physical violence, had an object thrown at them, punched, kicked or, beaten) over their lifetime due to their sexual orientation or gender identity or expression [2]. *Lifetime discrimination* was measured using a summed score (range: 0–15) of how often participants experienced five types of discrimination (e.g., not hired for a job, fired from a job) over their lifetime due to their sexual orientation or gender identity or expression [2]. *Day-to-day discrimination* was measured using a mean score of the 6-item scale (range: 0–5) in which participants were asking how often they experienced actions such as being treated with less courtesy and respect than other people, people acting as if they think you are not smart, receiving poorer service than other people at restaurants or stores. *Stigma* was measured using a summary score of an 8-item scale (range: 1–6) measuring positive and negative appraisal on their sexual or gender identity (positive identity questions were reverse coded) [2]. Higher scores indicate greater stigma. Participants were asked how much they disagreed/agreed with statements such as I feel ashamed of myself for being LGBT, I wish I weren’t LGBT, I feel comfortable being LGBT. *Loneliness* was measured using the three-item UCLA loneliness scale [54], in which participants were asked how often they felt they lacked companionship, felt left out, felt isolated from others with a summary score ranging from 0 to 4. Higher scores indicate greater loneliness.
Psychological and social resources	*Mastery* was measured using a mean of 4 items asking participants the extent to which they can accomplish things they want (1 = strongly disagree; 6 = strongly agree) [55]. *Partner status* was dichotomized as being married or partnered (= 1) or not being married or partnered (= 0). *Living alone* was dichotomized as currently living alone (=1) and not currently living alone (= 0). *Social network size* was measured by asking participants how many people (e.g., partner/spouse, ex-partner/spouse, friends, family members, neighbors) they have a close relationship with [56]. *Social support* was measured using an abbreviated 4-item version of the Social Support Instruments [57]. The summary score ranged from 1 to 4, with higher scores indicating greater social support. *Community engagement* was measured using a mean score of 4 items asking participants the extent to which they engage with or take part in the LGBT community (range: 1–6) with higher scores indicating greater community engagement [2].
Health-related indicators	Disability was dichotomized based on a six-item set of questions recommended by HHS Implementation Guidance on Data Collection Standards [58] to measure disability (0 = selected none of the six indicators, 1 = selected any of the indicators). *Physical functioning limitations* were measured using mean scores of 8 items asking participants how much difficulty they had with eight activities (e.g., a walking a quarter of a mile, reaching up over their head, stooping, bending or kneeling). *Cognitive impairment* was measured using mean scores of 6 items asking about difficulties in cognition. *Smoking* was dichotomized as 0 = not currently smoking 1 = currently smoking defined, as suggested by the CDC [59], as having smoked at least 100 cigarettes and currently smoking every day or some days. *Binge drinking* was dichotomized as 0 = maximum number of drinks at any drinking occasion is < 4 and 1 = maximum number of drinks >= 4. *Current reason for Medicare entitlement* was measured using CMS Medicare Beneficiary Summary File. A participant’s current reason for Medicare entitlement was either 0 = Old age and survivor’s insurance (OASI; entitlement based on age) or 1 = Disability insurance benefits (DIB; entitlement based on disability)
Socioeconomic factors	*Household income* was dichotomized to > 200% of the federal poverty guideline (FPG) = 0 and <= 200% of the FPG = 1 based on the federal poverty guidelines [60]. *Dual eligibility for Medicaid* was dichotomized as 0 = no months dually eligible for Medicaid during year and 1 = any months dually eligible for Medicaid during year. *Education level* was categorized as 1 = high school or less, 2 = some college, and 3 = graduate or professional degree. *Employment status* was dichotomized as 0 = not currently employed full- or part-time and 1 = currently employed full- or part-time
Background characteristics/ social location	Background characteristics/social location include *age* (in years), *gender* (1 = women, 2 = men, 3 = gender diverse), *gender identity* (1 = transgender, 2 = cisgender), *sexual identity* (1 = lesbian/gay, 2 = bisexual, 3 = sexual diverse), and *race/ethnicity* (1 = Hispanic, 2 = non-Hispanic White, 3 = Black or African American, 4 = Other [includes Asian, Native Hawaiian or Other Pacific Islander, American Indian, Alaskan Native, and multiracial])

Explanatory variables were number of and severity of chronic conditions (categorized into one of four groups), adverse experiences (lifetime victimization, lifetime discrimination, day-to-day discrimination, stigma, and loneliness); psychological and social resources (mastery, marital/partner status, living alone, social network size, social support, and community engagement); health-related indicators (disability, physical impairment, cognitive impairment, smoking, binge drinking, and current reason for Medicare entitlement); and socioeconomic factors (income, dual eligibility for Medicaid, employment, and education level).

### Analysis

First, descriptive statistics and bivariate analyses were conducted to explore the distributions of outcome and explanatory variables in the sample and across the four chronic conditions groups. For bivariate comparisons, one-way ANOVA was used for continuous variables and chi-square tests were used for categorical variables. Where significant differences were found at the pre-specified significance level of p < .05, post hoc analyses were conducted. Tukey’s Honest Significant Differences for multiple comparisons were used for ANOVA and multiple pairwise comparisons with Bonferroni adjusted p-values were used for chi-square. Then, a series of hierarchical linear and logistic regression models were estimated to examine the association between explanatory and outcome variables. Linear regression was used for continuous outcomes and logistic regression was used for binary outcomes. In the first model (Model 1), we examined the association between the four chronic conditions groups on each of the outcome variables: total Medicare spending, spending on physician services, high-cost beneficiary status, and healthcare utilization. In Model 2 we controlled for background characteristics/social location. In the final model (Model 3) we added and examined the remaining explanatory variables. Multicollinearity was assessed using Pearson’s Correlation Coefficient and variance inflation factor (VIF) and no issues were detected.

Linear models were used given their more straightforward interpretability and alignment with our research objectives for total Medicare spending and total spending on physician services. As such, we focus our main inference on this model. We also included a log-transformed model as a sensitivity check (see Supplementary S3–S5 Tables). This approach of using linear regression to model healthcare spending, while including an alternative specification for sensitivity analysis has been used previously [61,62]. A log-transformed transformation was used as the sensitivity analysis as prior research has shown that OLS on logged costs performs comparably to alternative estimators [63].

## Results

### Healthcare spending and utilization distributions

The average total Medicare spending was $11,671.47 (SD = 20,367.31), with a range from 0.00 to 165,650.02. The average spending on physician services was $893.46 (SD = 1,658.14), with a range from 0.00 to 14,158.42. Ninety-one participants were categorized as high-cost beneficiaries (highest 10% of Medicare spending). Finally, 43.13% of our sample (n = 389) reported using any healthcare services.

### Beneficiary characteristics

The distribution of background characteristics/social location by the four chronic conditions groups are presented in Table 2. The full, linked sample of CMS and NHAS consisted of 902 participants. Of these, 251 (27.83%) were part of the major complex chronic illness group, 252 (27.94%) were part of the minor complex chronic illness group, 132 (14.63%) were part of the simple chronic illness group, and 267 (29.60%) were part of the comparatively healthy group.

**Table 2:** Background characteristics/social location, key health indicators and risk and protective factors by severity of chronic conditions.

	Total Sample (n = 902)	Major Complex Chronic Illness (n = 251)	Minor Complex Chronic Illness (n = 252)	Simple Chronic Illness (n = 132)	Comparatively Health (n = 267)	Test of Significance χ2 or F test
	M (SD) or %	M (SD) or %	M (SD) or %	M (SD) or %	M (SD) or %	*p*
Background Characteristics/ Social Location						
Age, in years	69.61 (7.01)	70.08 (8.47) Range: 50–91	68.71 (7.09) Range: 50–87	70.30 (6.08) Range: 52–89	69.69 (5.68) Range: 53–89	p = .086
Gender						p = .209
Women	39.14	**	**	**	**	
Men	58.31	**	**	**	**	
Gender Diverse	2.55	**	**	**	**	
Gender Identity						p = .199
Cisgender	94.12	95.22	92.06	92.42	95.89	
Transgender	5.89	4.78	7.94	7.58	4.12	
Sexual Identity						p = .127
Lesbian/Gay	89.01	**	**	**	**	
Bisexual	7.77	**	**	**	**	
Sexual Diverse	3.22	**	**	**	**	
Race/Ethnicity						p = .307
Non-Hispanic White	83.56	78.89	82.47	89.39	86.09	
Hispanic	5.00	6.37	5.18	**	4.89	
Black or African American	6.11	7.57	7.17	**	4.14	
Other	5.33	7.17	5.18	**	4.89	
Adverse Experiences						
Lifetime Victimization	4.49 (5.21)	5.10 (6.19)	4.43 (4.85)	3.86 (4.53)	4.28 (4.82)	p = .125
Lifetime Discrimination	1.39 (2.55)	1.72 (3.15)	1.27 (2.10)	1.29 (2.57)	1.26 (2.28)	p = .134
Day-to-day Discrimination	0.74 (0.77)	0.86 (0.90)	0.73 (0.70)	0.74 (0.82)	0.65 (0.64)	p = .017^a^
Stigma	1.62 (0.77)	1.69 (0.79)	1.61 (0.80)	1.56 (0.73)	1.61 (0.73)	p = .422
Loneliness	1.56 (1.06)	1.74 (1.16)	1.60 (1.03)	1.40 (0.99)	1.45 (1.01)	p = .004^a,b^
Psychological and Social Resources						
Mastery	4.49 (0.92)	4.30 (0.97)	4.54 (0.91)	4.48 (0.93)	4.63 (0.84)	p < .001^a^
Marital/Partner Status						p = .475
Partnered	45.32	43.20	43.20	50.38	46.82	
Not Partnered	54.68	56.80	56.80	49.62	53.18	
Living Alone						p = .103
No	46.44	41.37	44.44	50.00	51.32	
Yes	53.56	58.63	55.56	50.00	48.68	
Social Network Size	8.45 (5.89)	7.51 (5.43)	8.37 (5.69)	8.73 (6.28)	9.29 (6.19)	p = .007^a^
Social Support	2.77 (1.05)	2.63 (1.06)	2.74 (1.02)	2.81 (1.09)	2.89 (1.04)	p = .036^a^
Community Engagement	4.04 (1.24)	3.93 (1.23)	3.98 (1.28)	4.10 (1.22)	4.18 (1.21)	p = .090
Health-Related Indicators						
Disability						p = .003^a,b^
No	51.58	44.13	47.62	61.72	56.49	
Yes	48.42	55.87	50.40	38.28	43.51	
Physical Impairment	0.82 (0.78)	1.08 (0.82)	0.77 (0.75)	0.65 (0.75)	0.69 (0.72)	p < .001^a,b,c^
Cognitive Impairment	15.13 (15.52)	19.06 (16.74)	15.82 (15.66)	11.34 (13.20)	12.68 (14.38)	p < .001^a,b^
Smoking						p = .076
No	94.23	91.77	93.15	**	96.17	
Yes	5.77	20.24	6.85	**	3.83	
Binge Drinking						p = .297
No	87.37	86.35	84.86	88.55	90.15	
Yes	12.63	13.65	15.14	11.45	9.85	
Current Reason for Medicare Entitlement						p < .001^a,b,d^
Age	84.37	76.89	78.97	92.42	92.51	
Disability	15.63	23.11	21.03	7.58	7.49	
Socioeconomic Factors						
Income						p = .002^a,d^
> 200% FPG	64.70	57.83	60.71	70.45	72.07	
=< 200% FPG	35.30	42.17	39.29	29.55	27.92	
Dually Eligible for Medicaid	17.74	32.27	19.84	8.33	6.74	p < .001^a,b,c,d,e^
Employment						p < .001^a,b,d^
Not Currently Employed	73.99	83.47	77.02	70.77	63.91	
Currently Employed	26.00	16.53	22.98	29.23	36.09	
Education						p = .126
High School or Less	7.33	10.76	8.00	**	4.87	
Some College	43.44	43.43	44.80	**	43.45	
Graduate or Professional Degree	49.22	45.82	47.20	**	51.69	

Notes. FPG = federal poverty guideline; Chi-squared or Fisher exact tests or ANOVA were conducted to examine differences by chronic conditions group; percentages for categorical variables and means with standard deviations in parentheses for continuous variables are reported. To ensure compliance with the CMS Data Use Agreement (DUA) requirements, all cells with n < 10, including any related percentages that could allow back-calculation, are suppressed.

^a^Value for major complex chronic illness group is significantly different from comparatively healthy group.

^b^Value for major complex chronic illness group is significantly different from simple chronic illness group.

^c^Value for major complex chronic illness group is significantly different from minor complex chronic illness group.

^d^Value for minor complex chronic illness group is significantly difference from comparatively healthy group.

^e^Value for minor complex chronic illness group is significantly difference from simple chronic illness group.

The average age of the 902 participants was 69.61 years (SD: 7.01; range: 50–91 years). Over half of the sample were men (58.31%), with 39.14% women, and 2.55% gender diverse. The sample comprised 5.89% (n = 53) who identified as transgender. The majority of the sample (88.01%) identified as lesbian or gay. For race and ethnicity, the sample was 83.56% non-Hispanic White, 6.11% Black or African American, 5.00% Hispanic, and 5.33% other (Asian, Native Hawaiian or Other Pacific Islander, American Indian or Alaskan Native, multiracial, and other). There were no significant differences in age, gender, gender identity, sexual identity, and race and ethnicity among the four chronic conditions groups. However, the proportion of participants who identified as non-Hispanic White was highest in the simple chronic illness group (89.39%), while the proportion of participants who identified as Black or African American, Hispanic, or other was highest in the major complex chronic illness group (7.57%, 6.37%, and 7.17%, respectively).

In terms of adverse experiences, the average lifetime victimization score was 4.49 (SD: 5.21), the average lifetime discrimination score was 1.39 (SD: 2.55), the average day-to-day discrimination score was 0.74 (SD: 0.77), the average stigma score was 1.62 (SD: 0.77), and loneliness was 1.56 (SD: 1.06). ANOVA tests showed no significant difference in lifetime victimization score, lifetime discrimination score or stigma across the four chronic conditions groups. There were significant differences in day-to-day discrimination and loneliness scores across the four chronic conditions groups. Post-hoc analyses showed day-to-day discrimination was higher on average for individuals in the major complex chronic illness group compared to those who were comparatively healthy (*p* = .008; 95% CI = [−0.39, −0.04]). For loneliness, individuals in the major complex chronic illness group had significantly higher loneliness scores compared to those in the simple chronic illness group (*p =* .015; 95% CI = [−0.63, −0.05]) and the comparatively healthy group (*p =* .012; 95% CI = [−0.52, −0.05]). In terms of psychological and social resources, a little over half of the sample was not partnered (54.68%) and living alone (53.56%). The average community engagement score was 4.04 (SD: 1.24). There were significant differences in terms of mastery, social network size, and social support. Those in the major complex chronic illness group had significantly lower mastery scores (*p* < .001; 95% CI = [0.13, 0.54]) and perceived social support (*p* = .023; 95% CI = [.03,.50]) and had the fewest people in their social networks on average (*p =* .003; 95% CI = [0.45, 3.11]) compared to those who were comparatively healthy.

There were significant differences across the four groups for most of the health-related indicators. Those in the major complex chronic illness group had a higher number of physical impairments compared to those in the minor complex chronic illness (*p* < .001; 95% CI = [−0.49, −0.14]), simple chronic illness (*p* < .001; 95% CI = [−0.65, −0.22]) and comparatively healthy (*p* < .001; 95% CI = [−0.56, −0.22]) groups. Respondents in the major complex chronic illness group reported significantly greater cognitive impairment compared to those in the simple chronic illness (*p* < .001; 95% CI = [−11.97, −3.48]) and comparatively healthy (*p* < .001; 95% CI = [−9.85, −2.91]) groups. Those in the major complex chronic illness group were more likely to report having a disability than those in the simple chronic illness (p = .011) and comparatively healthy (p = .042) groups. In terms of current reason for Medicare entitlement, the proportion of participants who received Medicare due to disability, as opposed to age was significantly greater in the major complex chronic illness and minor complex chronic illness groups, compared to the comparatively healthy group (*p* < .001 and *p* = .010, respectively). The majority of respondents reported not smoking (94.23%) or binge drinking (87.37%). There were no significant differences in binge drinking and smoking by chronic conditions group.

Regarding socioeconomic factors, those in the major complex chronic illness group were significantly more likely to be dually eligible for Medicaid compared to those in the minor complex chronic illness (*p* = .013), simple chronic illness (*p* < .001), and comparatively healthy (*p* < .001) groups. Those in the minor chronic illness group were also more likely to be dually eligible for Medicaid compared to those in the simple chronic illness (*p* = .032) and comparatively healthy groups (*p* < .001). Respondents in the major and minor complex illness groups were significantly more likely to be under the 200% FPG compared to those in the comparatively healthy group (*p* = .005 and *p* = .049, respectively). Finally, those in the major and minor complex chronic illness groups were less likely to be employed compared to those in the comparatively healthy groups (*p* < .001 and *p* = .010, respectively). Those in the major complex chronic illness group were also less likely to be employed than those in the simple chronic illness group (*p* = .036). There were no significant differences in terms of education level.

### Association between chronic conditions and healthcare spending and utilization

In Table 3 we present the results of multiple linear (total Medicare spending and spending on physician services) and logistic (high-cost beneficiaries and healthcare utilization) regressions on the chronic conditions group. As shown in the first model, those in the major complex chronic illness and minor complex chronic illness groups had significantly higher total Medicare spending compared to the comparatively healthy group. Beneficiaries in the simple chronic illness group did not have significantly higher total Medicare spending compared to those in the comparatively healthy group. All three chronic conditions groups had significantly higher spending on physician services than the comparatively healthy group. Beneficiaries in the major complex chronic illness and minor complex chronic illness groups were at increased odds for being a high-cost beneficiary compared to the comparatively healthy group. For healthcare utilization, beneficiaries in the major complex chronic illness, minor complex chronic illness, and simple chronic illness were significantly more likely to have any healthcare utilization compared to those in the comparatively healthy group.

**Table 3:** Bivariate linear and logistic regression of total Medicare spending, total physician spending, high-cost beneficiary status and healthcare utilization on chronic conditions (N = 902).

	Total Medicare Spending			Total Spending on Physician Services			High-Cost Beneficiary			Healthcare Utilization		
	β	95% CI	*p*	β	95% CI	*p*	OR	95% CI	*p*	OR	95% CI	*p*
Chronic Conditions Group												
Major Complex Chronic Illness	18,177.76	(14,898.26, 21,457.26)	<.001	2,110.96	(1,863.00, 2,358.92)	<.001	7.67	(3.88, 16.99)	<.001	141.56	(64.92, 373.22)	<.001
Minor Complex Chronic Illness	8,459.34	(5,183.20, 11,735.48)	<.001	833.52	(585.82, 1,081.23)	<.001	3.16	(1.49, 7.28)	.004	51.00	(23.76, 132.94)	<.001
Simple Chronic Illness	1,573.71	(−2,396.27, 5,541.69)	.438	402.40	(102.32, 702.49)	.009	0.90	(0.24, 2.81)	.857	31.07	(13.89, 83.15)	<.001

Note. OR = odds ratio; CI = confidence interval.

Next, we repeated these regressions, controlling for background characteristics/social location (Table 4). We found that controlling for age, gender, sexual identity, gender identity, and race and ethnicity, there were no changes in the relationships between chronic conditions group and total Medicare spending, spending on physician services, healthcare utilization, and high-cost beneficiary status. Those in the major complex chronic illness and minor complex chronic illness groups were associated with higher total Medicare spending and spending on physician services compared to those in the comparatively healthy group. Beneficiaries in the simple chronic illness group were also significantly associated with higher spending on physician services and healthcare utilization compared beneficiaries in the comparatively healthy group.

**Table 4:** Linear and logistic regressions of healthcare spending and utilization on chronic conditions and background characteristics/social location.

	Total Medicare Spending			Total Spending on Physician Services			High-Cost Beneficiary			Healthcare Utilization		
	β	95% CI	*p*	β	95% CI	*p*	AOR	95% CI	*p*	AOR	95% CI	*p*
Chronic Conditions Group												
Major Complex Chronic Illness	17,996.58	(14,854.30, 21,138.85)	<.001	2,143.95	(1,895.93, 2,391.97)	<.001	8.14	(4.00, 18.39)	<.001	161.70	(73.38, 429.61)	<.001
Minor Complex Chronic Illness	7,781.50	(4,634.99, 10,928.01)	<.001	856.90	(608.54, 1,105.25)	<.001	2.84	(1.31, 6.67)	.011	52.56	(24.35, 137.50)	<.001
Simple Chronic Illness	2,457.05	(−1,354.14, 6,268.24)	.207	408.04	(107.22, 708.85)	.008	0.93	(0.24, 2.99)	.904	32.98	(14.64, 88.69)	<.001
Comparatively Healthy	Referent											
Background Characteristics/ Social Location												
Age	−711.22	(−885.71, −536.72)	<.001	−16.56	(−30.33, −2.78)	.019	0.91	(0.88, 0.94)	<.001	0.96	(0.94, 0.99)	.003
Gender												
Women	Referent											
Men	5,988.70	(3,498.60, 8,478.79)	<.001	−129.34	(−325.89, 67.20)	.197	3.85	(2.15, 7.31)	<.001	0.64	(0.45, 0.91)	.014
Gender Diverse	−3,765.60	(−13,210.71, 5,679.52)	.435	−499.46	(−1,244.96, 246.05)	.190	0.60	(0.03, 5.55)	.688	0.60	(0.16, 2.13)	.429
Sexual Identity												
Lesbian/gay	Referent											
Bisexual	−919.76	(−5,508.30, 3,668.78)	.695	−252.82	(−615.00, 109.36)	.172	1.01	(0.32, 2.65)	.979	1.34	(0.70, 2.65)	.387
Sexual Diverse	7,760.30	(368.00, 15,152.61)	.040	−45.91	(−629.39, 537.57)	.878	5.90	(1.72, 19.63)	.004	0.87	(0.32, 2.42)	.791
Gender Identity												
Transgender	−385.63	(−7,046.34, 6,275.07)	.910	−93.93	(−619.66, 431.81)	.726	0.67	(0.14, 2.54)	.581	1.19	(0.47, 3.18)	.727
Race/Ethnicity												
Non-Hispanic White	Referent											
Hispanic	3,446.88	(−2,051.82, 8,945.58)	.220	−321.16	(−755.17, 112.86)	.147	1.24	(0.47, 2.96)	.638	1.18	(0.55, 2.66)	.677
Black or African American	−2,078.90	(−7,217.69, 3,059.90)	.428	−581.44	(−987.04, −175.83)	.005	0.55	(0.20, 1.34)	.211	0.71	(0.36, 1.42)	.325
Other	4,047.47	(−1,416.27, 9,511.21)	.147	77.47	(−353.79, 508.73)	.725	0.84	(0.29, 2.16)	.737	1.04	(0.48, 2.32)	.927

Note. AOR = adjusted odds ratio; CI = confidence interval.

Age was significantly, negatively associated with total Medicare spending, spending on physician services, high-cost beneficiary status, and healthcare utilization. Meaning, as age increased there was a decrease in total Medicare spending (β *=* −711.22; p < .001), spending on physician services (β *=* −16.56; p = .019) and decreased odds in being a high-cost beneficiary (adjusted odds ratio [AOR] = 0.91; p < .001) and healthcare utilization (AOR = 0.96; p = .003). Men had significantly higher total Medicare spending (β *=* 5,988.70; p < .001) and were more likely to be a high-cost beneficiary (AOR = 3.85; p < .001) compared to women, while they were less likely to use healthcare services (AOR = 0.64; p = .014). Sexually diverse individuals had significantly higher total Medicare spending (β = 7,760.30; p = .040) and were more likely to be considered a high-cost beneficiary (AOR = 5.90; p = .004) compared to those who were lesbian or gay. There were no significant differences between women and gender diverse participants and bisexual and lesbian or gay participants. Black or African American beneficiaries had significantly lower spending on physician services compared to non-Hispanic White beneficiaries (β = −581.44; p = .005), but not for total Medicare spending. There were no other significant differences in spending or utilization between beneficiaries of different racial and ethnic identities.

Finally, we conducted linear and logistic regression with a full model including background characteristics/social location, adverse experiences, psychological and social resources, health-related indicators, and socioeconomic factors (Table 5). As in the previous models, beneficiaries in the major complex chronic illness and minor complex chronic illness groups had significantly higher total Medicare spending and spending on physician services compared to beneficiaries in the comparatively healthy group. Those in the simple chronic illness group had significantly higher spending on physician serices (β = 415.66; p = .009) compared to those in the comparatively healthy group. Only those in the major complex chronic illness group were at increased odds of being a high-cost beneficiary compared to those in the comparatively healthy group (AOR = 4.58; p < .001). Beneficiaries in the major complex chronic illness, minor complex chronic illness, and simple chronic illness groups had increased odds of healthcare utilization compared to individuals in the comparatively healthy group.

**Table 5:** Linear and logistic regressions of healthcare spending and utilization on chronic conditions, background characteristics/social location, adverse experiences, psychological and social resources, health-related indicators, and socioeconomic factors.

	Total Medicare Spending			Total Spending on Physician Services			High-Cost Beneficiary			Healthcare Utilization		
	β	95% CI	*p*	β	95% CI	*p*	AOR	95% CI	*p*	AOR	95% CI	*p*
Chronic Conditions Group												
Major Complex Chronic Illness	14,165.96	(10,710.95, 17,620.98)	<.001	2,144.13	(1,865.82, 2,422.44)	<.001	4.58	(2.05, 11.11)	<.001	221.67	(91.09, 669.95)	<.001
Minor Complex Chronic Illness	6,192.75	(2,922.73, 9,462.78)	<.001	860.88	(597.48, 1,124.29)	<.001	1.82	(0.77, 4.53)	.182	68.39	(29.31, 200.53)	<.001
Simple Chronic Illness	2,787.67	(−1,083.94, 6,659.28)	.159	415.66	(103.80, 727.53)	.009	0.72	(0.17, 2.51)	.627	40.17	(16.62, 120.26)	<.001
Background Characteristics/ Social Location												
Age	−366.97	(−620.33, −113.61)	.005	−34.70	(−55.11, −14.30)	.001	0.95	(0.89, 1.00)	.068	0.93	(0.89, 0.96)	<.001
Gender												
Men	5,364.94	(2,550.76, 8,179.11)	<.001	−69.27	(−295.96, 157.42)	.549	4.52	(2.20, 10.04)	<.001	0.75	(0.49, 1.14)	.174
Gender Diverse	−1,685.32	(−11,298.07, 7,927.42)	.731	−600.04	(−1,374.37, 174.29)	.129	0.73	(0.02, 9.16)	.826	0.42	(0.10, 1.65)	.214
Sexual Identity												
Bisexual	−4,250.29	(−9,036.32, 535.74)	.082	−352.27	(−737.80, 33.26)	.074	0.48	(0.12, 1.48)	.239	1.09	(0.52, 2.33)	.813
Sexual Diverse	1,205.43	(−6,632.00, 9,042.86)	.763	−163.65	(−794.97, 467.67)	.612	1.82	(0.34, 8.57)	.464	0.62	(0.19, 1.93)	.409
Gender Identity												
Transgender	1,045.09	(−5,871.55, 7,961.72)	.767	49.70	(−507.46, 606.85)	.861	1.30	(0.25, 5.20)	.731	1.95	(0.69, 5.92)	.219
Race/Ethnicity												
Hispanic	288.92	(−5,592.14, 6,619.99)	.923	−242.80	(−716.57, 230.89)	.315	0.90	(0.27, 2.60)	.851	0.94	(0.39, 2.38)	.901
Black or African American	−4,066.24	(−9,578.60, 1,446.13)	.149	−560.10	(−1,004.13, −116.07)	.014	0.41	(0.13, 1.17)	.112	0.65	(0.30, 1.42)	.277
Other	2,111.48	(−3,568.01, 7,790.96)	.466	127.88	(−329.61, 585.38)	.584	0.69	(0.20, 2.05)	.534	1.15	(0.49, 2.77)	.749
Adverse Experiences												
Lifetime Victimization	185.32	(−116.75, 487.38)	.230	−10.91	(−35.24, 13.42)	.380	1.04	(0.98, 1.10)	.243	1.00	(0.95, 1.04)	.919
Lifetime Discrimination	−209.87	(−829.33, 409.59)	.507	−25.53	(−75.43, 24.37)	.316	0.97	(0.85, 1.09)	.575	0.95	(0.87, 1.04)	.261
Day-to-day Discrimination	−2,542.01	(−4,601.57, −482.44)	.016	51.77	(−114.13, 217.67)	.541	0.73	(0.47, 1.11)	.159	1.19	(0.88, 1.63)	.268
Stigma	620.51	(−1,144.50, 2,385.51)	.491	97.71	(−44.46, 239.89)	.178	1.05	(0.74, 1.47)	.787	0.95	(0.73, 1.22)	.668
Loneliness	−407.21	(−2,086.02, 1,271.60)	.635	41.51	(−93.72, 176.74)	.548	0.91	(0.64, 1.28)	.582	1.00	(0.78, 1.28)	.991
Psychological and Social Resources												
Mastery	24.53	(−1,547.50, 1,596.56)	.976	−5.81	(−132.44, 120.82)	.928	1.05	(0.76, 1.46)	.791	1.10	(0.87, 1.38)	.437
Marital/Partner Status – Partnered	2,122.88	(−1,459.98, 5,705.74)	.246	113.40	(−175.21, 402.00)	.442	1.03	(0.45, 2.31)	.948	1.19	(0.69, 2.08)	.529
Living alone – Yes	3,280.96	(−13.53, 6,575.44)	.051	124.67	(−140.71, 390.05)	.358	1.81	(0.88, 3.81)	.111	1.44	(0.88, 2.39)	.152
Social network size	−145.93	(−390.61, 98.75)	.243	−6.47	(−26.18, 13.24)	.520	0.98	(0.92, 1.04)	.554	1.02	(0.99, 1.06)	.221
Social support	−7.69	(−1,718.53, 1,703.15)	.993	5.40	(−132.41, 143.21)	.939	0.98	(0.68, 1.41)	.917	1.07	(0.83, 1.37)	.607
Community Engagement	139.08	(−1,010.75, 1,288.92)	.813	−27.80	(−120.42, 64.82)	.557	0.84	(0.65 1.08)	.172	0.99	(0.84, 1.17)	.903
Health-Related Indicators												
Disability – Yes	3,682.43	(808.62, 6,556.23)	.012	54.61	(−176.89, 286.10)	.644	1.77	(0.94, 3.38)	.080	1.02	(0.67, 1.56)	.922
Physical Impairment	280.66	(−1,690.92, 2,252.25)	.780	−9.49	(−168.30, 149.33)	.907	1.12	(0.74, 1.68)	.574	0.99	(0.74, 1.32)	.928
Cognitive Impairment	55.09	(−45.17, 155.35)	.282	0.50	(−7.58, 8.58)	.903	1.01	(0.99, 1.03)	.282	1.01	(0.99, 1.02)	.461
Smoking – Yes	8,869.36	(3,146.64, 14,592.08)	.003	32.88	(−428.10, 493.86)	.889	2.24	(0.87, 5.57)	.087	0.83	(0.37, 1.88)	.644
Binge Drinking – Yes	−4,937.15	(−8,610.80, −1,263.49)	.009	−308.81	(−604.73, −12.89)	.041	0.51	(0.22, 1.10)	.103	0.87	(0.52, 1.46)	.593
Current Reason for Medicare Entitlement – Disability	7,154.10	(2,083.23, 12,224.97)	.006	−266.81	(−675.29, 141.66)	.201	1.86	(0.71, 4.84)	.203	0.45	(0.21, 0.96)	.040
Socioeconomic Factors												
Income, =< 200% FPG	−2,999.28	(−6,148.54, 149.97)	.062	−335.11	(−588.79, −81.43)	.010	0.77	(0.38, 1.53)	.468	0.80	(0.50, 1.27)	.340
Dually Eligible for Medicaid -Enrolled	10,076.35	(5,806.61, 14,346.09)	<.001	150.79	(−193.15, 494.73)	.390	2.31	(1.08, 4.98)	.031	1.25	(0.67, 2.34)	.485
Currently Employed	894.12	(−2,078.63, 3,866.87)	.556	−107.21	(−346.67, 132.25)	.381	1.14	(0.56, 2.27)	.705	0.79	(0.51, 1.23)	.299
Education												
Some College	1,329.97	(−3,705.30, 6,365.24)	.605	262.51	(−143.09, 668.12)	.205	0.95	(0.40, 2.36)	.914	1.15	(0.57, 2.34)	.694
Graduate or Professional Degree	516.90	(−4,746.40, 5,780.19)	.847	250.94	(−173.03, 674.91)	.246	0.93	(0.36, 2.53)	.889	1.56	(0.74, 3.28)	.239

Note. AOR = adjusted odds ratio; CI = confidence interval; FPG = federal poverty guideline.

Age remained significantly, negatively associated with total Medicare spending (p = .005), spending on physician services (p = .001), and healthcare utilization (p < .001), but not being a high-cost beneficiary. Compared to women, men had significantly higher total Medicare spending (β = 5,364.94; p < .001) and greater odds of being a high-cost beneficiary (AOR = 4.52; p < .001). There were no significant differences in total Medicare spending, spending on physician services, high-cost beneficiary status or healthcare utilization between women and gender diverse participants or across sexual identities and gender identity. There were no significant differences in total Medicare spending by race and ethnicity. However, Black or African American beneficiaries continued to have significantly lower spending on physician services compared to non-Hispanic White beneficiaries (β *=* −560.10; p = .014). There were no significant differences in high-cost beneficiary status or healthcare utilization by race and ethnicity.

Among social risk factors, as frequency of day-to-day discrimination increased, there was a significant decrease in total Medicare spending (β = −2,542.01; p = .016). There were no significant relationships between other social risk factors and spending on physician services, high-cost beneficiary status, and healthcare utilization. There were no significant elationships between psychological and social resources and total Medicare spending, spending on physician services, high-cost beneficiary status and healthcare utilization.

Having a disability was consistent with higher total Medicare spending (β *=* 3,682.43; p = .012) compared to those without a disability, but there was no significant association with spending on physician services, being a high-cost beneficiary or healthcare utilization. Participants who reported currently smoking had significantly higher total Medicare spending (β *=* 8,869.36; p = .003) compared to those not smoking. Conversely, individuals reporting binge drinking had significantly lower total Medicare spending (β = −4,937.15; p = .008) and spending on physician services (β = −308.81; p = .041). There was no significant association between binge drinking and being a high-cost beneficiary and healthcare utilization. Participants whose current reason for Medicare entitlement was due to disability had significantly higher total Medicare spending compared to those whose Medicare entitlement was due to age (β = 7,154.10; p = .006) and may reflect lower odds of healthcare utilization (AOR = 0.45; p = .040). Reason for Medicare entitlement was not significantly associated with spending on physician services or being a high-cost beneficiary.

Beneficiaries who had incomes equal to or below the 200% FPG were associated with lower spending on physician services (β *=* −335.11; p = .010) compared to those above 200% FPG. Those who were dually eligible for Medicaid and Medicare were associated with higher total Medicare spending (β *=* 10,076.35; p < .001) and were more likely to be a high-cost beneficiary (AOR = 2.31; p = .030). There was no significant association between employment or education and healthcare spending or utilization.

### Sensitivity analysis

Results from the sensitivity analysis (Supplementary S3–S5 Tables) were overall consistent with the findings from the linear regression. However, some coefficient estimates differed in significance. In the sensitivity analysis, age and identifying as a man versus women were no longer significantly associated with total Medicare spending, although the direction of the relationship remained consistent with the linear regression. Black participants were also more likely to have both lower total Medicare spending and spending on physician services. Day-to-day discrimination, smoking, and reason for Medicare entitlement were no longer significantly associated with total Medicare spending. However, the direction of the relationship between total Medicare spending and day-to-day discrimination and reason for Medicare entitlement remained consistent with the linear regression. The sensitivity analyses also found employment was associated with lower total Medicare spending. In regard to spending on physician services, loneliness was significantly negatively associated with spending, while binge drinking was no longer associated with spending.

## Discussion

Understanding the relationship between health disparities in chronic health conditions, and its association to healthcare utilization and cost among underserved populations is critical given the need to promote health equity and patient-centered care models while ensuring cost-effectiveness [64]. Medicare spending accounted for 21% of national health spending [65], and projections indicate that Medicare spending is growing at an increasing rate compared to previous years. This has been attributed to growing enrollment in Medicare, increasing service utilization, and rising costs in healthcare [66]. This high and growing cost of healthcare, combined with the imperative to ensure the healthcare system meets the needs of all beneficiaries, are of great concern to policymakers and are prominent in discussions of national priorities [64]. Identifying the driving factors of healthcare cost and utilization within underserved at-risk populations, such as SGM older adults, is a necessary step to address these concerns.

Among the general population as the number or complexity of chronic conditions increases so does spending on healthcare [67,68]. Given that SGM older adults have higher risks of certain chronic conditions and multimorbidity [5], understanding how chronic conditions affect healthcare use and spending among SGM older adults will be consequential as policymakers seek to reduce healthcare cost. First, we found that SGM older adults with the greatest severity and complexity of chronic conditions were more likely to have a disability, be dually eligible for Medicaid, have Medicare with entitlement due to disability, more physical and cognitive impairment, more likely to experience day-to-day discrimination and loneliness, with less mastery, social support, and smaller social network size, and were more likely to be low-income and unemployed, compared to those who were comparatively healthy.

Overall, we found greater chronic condition complexity was associated with greater healthcare spending and utilization. SGM beneficiaries in the major complex chronic conditions group, when compared to the other relatively healthy groups, had significantly higher total Medicare spending and spending on physician services and higher odds of being a high-cost beneficiary and more healthcare utilization. Similarly, those in the minor complex chronic group had significantly higher total Medicare spending and physician spending and had higher odds of using healthcare services but were not more likely to be a high-cost beneficiary compared to those in the comparatively healthy group. These findings were consistent with existing literature on chronic conditions and healthcare spending among older adults in general [69]. In one systematic literature review of healthcare spending and utilization by older adults with multiple chronic conditions, all but three of 35 studies found a positive association between the presence of multiple chronic conditions and healthcare expenditures [69]. Our findings also align with prior research on healthcare utilization, which has shown an increase in the use of primary care services for older adults with greater medical complexity and more chronic conditions [70].

Those in the simple chronic conditions group in this study had greater odds of healthcare utilization and higher physician spending compared to those in the comparatively healthy group. This is likely to indicate an increase in practices of health maintenance, such as seeing a primary care physician for health maintenance services or preventive care. Conversely, those in the simple chronic group were not more likely to be a high-cost beneficiary compared to those in the comparatively healthy group. This points to areas of potential intervention for reducing healthcare spending. For example, timely detection of risk factors utilizing clinical tools and care coordination interventions have the potential to enhance health outcomes by promoting the prevention of progression to multimorbidity [71]. As such, future research should examine SGM access to and use of care coordination and its potential subsequent effects on chronic conditions management.

Dual eligible beneficiaries overall tend to report worse health compared to non-dual beneficiaries. In 2024, only 25% of dual eligible beneficiaries over 65 reported their health status as being “excellent or very good” compared to 53% of non-dual Medicare beneficiaries. Additionally, 42% of dually eligible beneficiaries over 65 reported at least one limitation with ADLs compared to 18% of non-dual Medicare beneficiaries and had higher Medicare spending on inpatient hospitalizations, skilled nursing facility, home health, other outpatient services, and prescription drugs compared to Medicare only beneficiaries [72]. Given the relationship between dual eligibility and disability, our findings suggest higher total Medicare spending is closely linked to disability. Consistent with this literature, we find that SGM older adults living with disability and dually eligible for Medicaid had greater total Medicare spending. This could indicate SGM older adults with disability face additional financial burdens related to healthcare spending. As higher risks of disability among SGM older adults have been documented [5,73], it is imperative to develop more targeted policies designed to reduce financial burden, such as improving access to preventative care and making low cost services (e.g., telehealth services) available and accessible for SGM older adults with disability. Additional research on reducing healthcare spending for dually eligible beneficiaries has suggested attending to social determinants of health, such as housing, food insecurity, and social isolation [74], all key risk factors for SGM older adults [75,76].

Behavioral health factors were associated with the cost of healthcare for SGM beneficiaries. Those who reported smoking had significantly greater total Medicare spending compared to those who did not smoke in the linear regression. This finding is in line with previous work linking smoking and increased healthcare spending among older adults [26]. Conversely, total Medicare spending was lower for SGM participants who reported binge drinking. Among the general older adult population, studies on the relationship between binge drinking and healthcare have focused on utilization. Older adults who report binge drinking are more likely to use the ED [29,77] and less likely to use physician services [27]. Both excessive drinking and smoking can have harmful effects on health outcomes and mortality [78,79]. For instance, smoking, particularly heavy smoking compared to light or non-smoking, was associated with increased frailty and poorer health status at older ages [80]. This finding is concerning given the higher rates of smoking among sexual minority older adults compared to their heterosexual counterparts. SGM binge drinkers, on the other hand, may have lower Medicare spending, as a result of chronic stress and the avoidance of care. More research is needed on the relationship between behavioral health and healthcare cost and use, particularly on the impact of smoking given our inconsistent findings in the sensitivity analysis.

Discrimination of SGM individuals in healthcare has been reported extensively, and as a result SGM older adults may be less likely to utilize healthcare services, as they report distrust of healthcare providers and settings [81,82]. In this study, higher day-to-day discrimination was found to be associated with greater likelihood of being in the major complex chronic illness group and lower total Medicare spending in the linear regression, which may indicate decreased access to healthcare for SGM Medicare beneficiaries. Thus, blanketly expanding medical services may not benefit SGM older adults unless those medical services and providers are culturally competent and responsive to SGM health needs and contexts [83]. As such, there is a need for additional research on discrimination and healthcare spending, LGBTQ + -specific competencies, and increased medical training on how personal and professional attitudes and behaviors toward SGM populations affect healthcare provision [83].

Additionally, the relationship between wealth and health is concerning given that SGM older adults have faced a lifetime of discrimination in employment and earnings, with one third of SGM older adults living at or below 200% FPG [84–86]. Across the SGM population economic disparities have been documented [10]. Furthermore, those with multiple marginalized identities and social locations (e.g., SGM who are also racially minoritized) face additional barriers to care [87]. We found reduced spending on physician services for Black or African American SGM older adults. Low spending on physician services could indicate lower use of routine or preventive care. This is potentially problematic given the benefits of outpatient physician services, such as primary care, which has been shown to prevent illness and mortality [88]. Reduced use of routine or preventive physician services may lead to increased use of more expensive healthcare services such as acute and emergency care [88,89].

High-cost beneficiaries (those in the top 10% of total Medicare spending) were more likely to be in the major complex chronic illness group, to be men compared to women and to be dually eligible for Medicaid. There were important differences in variables associated with total Medicare spending and being a high-cost beneficiary. For instance, being in the minor complex chronic illness and simple chronic illness groups and having a disability were all significantly associated with greater total Medicare spending yet not being a high-cost beneficiary. It is only those in the major complex chronic illness group that had significantly greater spending and were more likely to be a high-cost beneficiary. These findings support existing research that has shown health status as a significant predictor of being a high-cost Medicare beneficiary among non-SGM populations [51,90]. Additional work on high-cost Medicare beneficiaries has found associations between higher spending and younger age and reason for Medicare entitlement being due to disability or End Stage Renal Disease [53,91]. This points to the importance of understanding the complexities in relationships between age, disability, chronic conditions, and healthcare spending and utilization. Younger or more disabled SGM participants may have differing chronic conditions compared to older participants, such as HIV, which can contribute to higher healthcare spending and utilization. For instance, the largest percentage of older adults living with HIV in the US in 2015 was among those age 50–54 years (38%) compared to all other older adults age groups [92].

We also found higher likelihood of healthcare utilization among participants who were in the major chronic illness, minor chronic illness, and simple chronic illness groups as compared to the comparatively healthy participants. Age had an inverse relationship with the likelihood of healthcare utilization. Interestingly, disability as the current reason for Medicare entitlement was associated with a lower likelihood of healthcare utilization, which was positively associated with higher total Medicare spending. These findings suggest that although SGM participants living with disability may be less likely to use healthcare services overall, when they do use healthcare, they are using more expensive healthcare services such as inpatient hospitalizations or skilled nursing facilities, thereby incurring higher cost.

Progovac et al. [47] found that transgender Medicare beneficiaries were more likely to have a disability than non-transgender beneficiaries. Additionally, they found greater use of hospitalization and emergency departments among transgender beneficiaries. Taken together, these findings lend support to the idea that SGM Medicare beneficiaries with disabilities may be more likely to use more expensive healthcare services. Interestingly, we found no difference between transgender and non-transgender beneficiaries in terms of healthcare utilization. Previous research on healthcare spending and utilization among transgender Medicare beneficiaries has identified transgender patients using diagnosis-code algorithms which identify beneficiaries in treatment with specified diagnoses, such as gender dysphoria, and paid for by Medicare related to their medical care, e.g., gender transition [47]. Our study uses self-identification of transgender identity, which can be considered a strength. However, transgender Medicare beneficiaries receiving healthcare services related to their transgender identity or care may differ from those not using healthcare services. Additional research is needed to better understand the complexities in identifying transgender beneficiaries and how that affects healthcare spending and utilization.

While chronic illness complexity was the only variable significantly associated with all healthcare spending and utilization outcomes in this analysis, other important subgroup differences did emerge. Lower spending on physician services, for example, was also associated with increased age, being Black or African American compared to non-Hispanic White and having low income. Men compared to women in this study had significantly higher odds of being a high-cost beneficiary. However, there was no significant difference between men and women in terms of healthcare utilization. This may indicate that SGM men could be using more high-cost services, such as hospitalizations or emergency room (ER) visits. Additionally, this finding that men were more likely than women to be high-cost beneficiaries contradicts existing literature of older adults in the general population. Prior research on older adults in general has found women spent more on healthcare and utilized healthcare more than men [93–95]. Gavulic and Gonzales [36] have found that men in same-sex relationships had higher spending on healthcare compared to men in different-sex relationships. They noted elevated spending on prescription drugs among men in same-sex relationships and speculated increased spending may be related to the treatment of sexually transmitted infections, which can be expensive. Additional research is needed to explore factors contributing to increased healthcare spending among SGM men.

Although previous research has noted SGM older adults face financial barriers to healthcare due to lower household incomes, high living expenses, lack of employment or potential job loss, and gender pay inequities compared to heterosexual adults [76], we did not find a significant association between healthcare utilization and employment, education, or income among SGM Medicare beneficiaries. Our findings suggest that chronic illness complexity and disability are key predictors of healthcare utilization as opposed to other socioeconomic factors. These findings are not consistent with existing literature, which has linked low income with lower healthcare utilization [21,22] and higher education to be associated with a lower probability of expensive healthcare, such as hospitalizations [23]. The coverage offered by Medicare may help to mitigate the cost-related barriers to care for this group of SGM older adults who have successfully enrolled. Once the initial barrier of insurance is resolved, healthcare utilization may be driven more directly by perceived health needs than by socioeconomic constraints.

### Limitations

While this is one of the first studies to examine SGM older adults’ health disparities using administrative health data, our findings must be interpreted in the context of the limitations of the study. First, while we used administrative health data to capture number of chronic conditions, we lacked information on severity for each condition. This could affect the relationships between chronic conditions and healthcare spending and utilization. Second, data on spending and utilization come from the Medicare Cost and Use file, meaning only spending on services covered by Medicare were included. We do not have spending or utilization data related to long-term services and supports, which are often covered by Medicaid. As such per beneficiary spending, particularly for dually eligible participants, may be underestimated. Additionally, there is the potential for other chronic conditions, e.g., HIV/AIDS, that are generally not included in the examination of chronic conditions and healthcare spending and utilization among Medicare beneficiaries to impact these findings. Future research in this area should examine in-depth the effects of other chronic conditions on healthcare and spending, particularly conditions such as HIV/AIDS, which are often stigmatized [96].

Findings from this study are not generalizable to the US SGM older adult population more broadly, as our sample includes only the 902 NHAS participants who consented to linkage with the CMS data. In addition, the voluntary nature of NHAS survey participation and possible reporting biases (e.g., recall or social desirability bias) could affect the observed associations reported in this study. Additional research is also needed to tease apart specific aspects of psychological and social resources, such as mastery as well as the size of social network, extent of social support, and levels of community engagement. Additionally, Medicare Cost and Use files cannot speak to the quality of interactions with healthcare services or providers, which is particularly important given what we know about stigma and discrimination in the healthcare system for SGM older adults. Future research is needed to examine the relationships between healthcare spending, utilization, and quality of care among underserved populations, including SGM older adults. Finally, additional research is needed that compares healthcare spending and utilization patterns over time as well as between Medicare SGM and non-SGM beneficiaries.

### Conclusion

This study examined the relationship between type and severity of chronic health conditions and healthcare spending and utilization among an underserved and understudied population. The study used linked data from Aging with Pride: National Health, Aging and Sexuality/Gender Study (NHAS) and CMS Chronic Conditions Warehouse data (n = 902) to examine chronic conditions, healthcare spending and utilization among a diverse sample of SGM older adult Medicare beneficiaries. By merging NHAS survey data with CMS administrative health data, our findings were considerably strengthened by identifying, actual service use as opposed to self-report, several modifiable factors that impact chronic condition complexity among SGM older adults.

By merging NHAS survey data with CMS administrative health data, our findings identified several modifiable factors that impact chronic condition complexity among SGM older adults. These included experiences of day-to-day discrimination, disability, physical and cognitive impairment, loneliness, feelings of mastery, social network size and support, income, employment, and dual eligibility for Medicare and Medicaid. The research found that chronic illness complexity and disability were key predictors of increased healthcare spending and utilization, which is concerning given SGM older adults face higher risk of experiencing disability, certain chronic conditions, and multimorbidity [5,97]. More research on the experiences of SGM older adults dually eligible for Medicaid and those living with disability is needed given their increased risk for high complexity in chronic conditions and high healthcare spending, yet at times lower healthcare utilization. Understanding the relationship between chronic health conditions and healthcare cost and utilization for underserved older adults in general as well as those in specific at-risk subgroups is a critical step in developing responsive health services and effective interventions to promote healthy aging in our increasingly diverse society.

## Supporting information

S1 Table CMS Cost and Use service claims used in Medicare spending and utilization. (DOCX)

S2 Table Classification of Chronic Conditions based on CMS Chronic Conditions and Other Chronic Conditions files. (DOCX)

S3 Table Bivariate linear regression of log-transformed total Medicare spending and total physician spending on chronic conditions (N = 902; exponentiated coefficients). (DOCX)

S4 Table Linear regressions of log-transformed healthcare spending on chronic conditions and background characteristics/social location. (DOCX)

S5 Table Linear regressions of log-transformed healthcare spending on chronic conditions, background characteristics/social location, adverse experiences, psychological and social resources, health-related indicators, and socioeconomic factors. (DOCX)

## References

[1] Administration for Community Living. 2021 Profile of Older Americans. 2023.

[2] Fredriksen-Goldsen KI, Kim H-J. The Science of Conducting Research With LGBT Older Adults- An Introduction to Aging with Pride: National Health, Aging, and Sexuality/Gender Study (NHAS). Gerontologist. 2017;57(suppl 1):S1–14. doi: 10.1093/geront/gnw212 28087791 PMC5241759

[3] National Institutes of Health. Minority health and health disparities strategic plan 2021-2025: Taking the next steps. Bethesda, MA: 2021.

[4] Healthy People 2030. Healthy People 2030: Social determinants of health 2024. [accessed November 30, 2024]. https://odphp.health.gov/healthypeople

[5] Fredriksen-Goldsen KI, Kim H-J, Shui C, Bryan AEB. Chronic Health Conditions and Key Health Indicators Among Lesbian, Gay, and Bisexual Older US Adults, 2013-2014. Am J Public Health. 2017;107(8):1332–8. doi: 10.2105/AJPH.2017.303922 28700299 PMC5508186

[6] Caceres BA, Travers J, Sharma Y. Differences in Multimorbidity among Cisgender Sexual Minority and Heterosexual Adults: Investigating Differences across Age-Groups. J Aging Health. 2021;33(5–6):362–76. doi: 10.1177/0898264320983663 33382014 PMC8122030

[7] Dyar C, Taggart TC, Rodriguez-Seijas C, Thompson RG Jr, Elliott JC, Hasin DS, et al. Physical Health Disparities Across Dimensions of Sexual Orientation, Race/Ethnicity, and Sex: Evidence for Increased Risk Among Bisexual Adults. Arch Sex Behav. 2019;48(1):225–42. doi: 10.1007/s10508-018-1169-8 29633061 PMC6382069

[8] Han BH, Duncan DT, Arcila-Mesa M, Palamar JJ. Co-occurring mental illness, drug use, and medical multimorbidity among lesbian, gay, and bisexual middle-aged and older adults in the United States: a nationally representative study. BMC Public Health. 2020;20(1):1123. doi: 10.1186/s12889-020-09210-6 32746891 PMC7401198

[9] Fredriksen-Goldsen KI, Cook-Daniels L, Kim H-J, Erosheva EA, Emlet CA, Hoy-Ellis CP, et al. Physical and mental health of transgender older adults: an at-risk and underserved population. Gerontologist. 2014;54(3):488–500. doi: 10.1093/geront/gnt021 23535500 PMC4013724

[10] Fredriksen Goldsen KI, Romanelli M, Hoy-Ellis CP, Jung H. Health, economic and social disparities among transgender women, transgender men and transgender nonbinary adults: Results from a population-based study. Prev Med. 2022;156:106988. doi: 10.1016/j.ypmed.2022.106988 35150748 PMC8954758

[11] Fredriksen-Goldsen KI, Emlet CA, Kim H-J, Muraco A, Erosheva EA, Goldsen J, et al. The physical and mental health of lesbian, gay male, and bisexual (LGB) older adults: the role of key health indicators and risk and protective factors. Gerontologist. 2013;53(4):664–75. doi: 10.1093/geront/gns123 23034470 PMC3709843

[12] Pharr JR. Health Disparities Among Lesbian, Gay, Bisexual, Transgender, and Nonbinary Adults 50 Years Old and Older in the United States. LGBT Health. 2021;8(7):473–85. doi: 10.1089/lgbt.2021.0009 34534016

[13] Guo Y, Li Q, Yang X, Jaffee MS, Wu Y, Wang F, et al. Prevalence of Alzheimer’s and Related Dementia Diseases and Risk Factors Among Transgender Adults, Florida, 2012‒2020. Am J Public Health. 2022;112(5):754–7. doi: 10.2105/AJPH.2022.306720 35324265 PMC9010917

[14] Dieleman JL, Cao J, Chapin A, Chen C, Li Z, Liu A, et al. US Health Care Spending by Payer and Health Condition, 1996-2016. JAMA. 2020;323(9):863–84. doi: 10.1001/jama.2020.0734 32125402 PMC7054840

[15] Institute of Medicine. Health status and health care service utilization. Retooling for an Aging America: Building the Health Care Workforce. National Academies Press (US); 2008. 25009893

[16] Jones CH, Dolsten M. Healthcare on the brink: navigating the challenges of an aging society in the United States. NPJ Aging. 2024;10(1):22. doi: 10.1038/s41514-024-00148-2 38582901 PMC10998868

[17] Cheng Y, Goodin AJ, Pahor M, Manini T, Brown JD. Healthcare Utilization and Physical Functioning in Older Adults in the United States. J Am Geriatr Soc. 2020;68(2):266–71. doi: 10.1111/jgs.16260 31755551

[18] Khavjou OA, Anderson WL, Honeycutt AA, Bates LG, Razzaghi H, Hollis ND, et al. National Health Care Expenditures Associated With Disability. Med Care. 2020;58(9):826–32. doi: 10.1097/MLR.0000000000001371 32826747 PMC7505687

[19] Pena MT, Mohamed M, Burns A, Cubanski J, Sroczynski B, Chidambaram P. Enrollment and spending patterns among Medicare-Medicaid enrollees (dual eligibles). Kaiser Family Foundation. 2023.

[20] MedPAC. Data book section 4: Dual-eligible beneficiaries. The Medicare Payment Advisory Commission; 2024.

[21] McMaughan DJ, Oloruntoba O, Smith ML. Socioeconomic Status and Access to Healthcare: Interrelated Drivers for Healthy Aging. Front Public Health. 2020;8:231. doi: 10.3389/fpubh.2020.00231 32626678 PMC7314918

[22] Woolf SH, Aron L, Dubay L, Simon SM, Zimmerman E, Luk KX. How Are Income and Wealth Linked to Health and Longevity? Urban Institute & Center on Society & Health; 2015.

[23] Yue D, Ponce NA, Needleman J, Ettner SL. The relationship between educational attainment and hospitalizations among middle-aged and older adults in the United States. SSM Popul Health. 2021;15:100918. doi: 10.1016/j.ssmph.2021.100918 34568538 PMC8449049

[24] Cameron KA, Song J, Manheim LM, Dunlop DD. Gender disparities in health and healthcare use among older adults. J Womens Health (Larchmt). 2010;19(9):1643–50. doi: 10.1089/jwh.2009.1701 20695815 PMC2965695

[25] Wallace J, Jiang K, Goldsmith-Pinkham P, Song Z. Changes in Racial and Ethnic Disparities in Access to Care and Health Among US Adults at Age 65 Years. JAMA Intern Med. 2021;181(9):1207–15. doi: 10.1001/jamainternmed.2021.3922 34309621 PMC8314180

[26] An R. Health care expenses in relation to obesity and smoking among U.S. adults by gender, race/ethnicity, and age group: 1998-2011. Public Health. 2015;129(1):29–36. doi: 10.1016/j.puhe.2014.11.003 25542741

[27] Jenkins KR, Zucker RA. The prospective relationship between binge drinking and physician visits among older adults. J Aging Health. 2010;22(8):1099–113. doi: 10.1177/0898264310376539 20693519 PMC4618665

[28] Merrick ESL, Hodgkin D, Garnick DW, Horgan CM, Panas L, Ryan M, et al. Older adults’ inpatient and emergency department utilization for ambulatory-care-sensitive conditions: relationship with alcohol consumption. J Aging Health. 2011;23(1):86–111. doi: 10.1177/0898264310383156 20935248 PMC3021178

[29] Han BH, Moore AA, Ferris R, Palamar JJ. Binge Drinking Among Older Adults in the United States, 2015 to 2017. J Am Geriatr Soc. 2019;67(10):2139–44. doi: 10.1111/jgs.16071 31364159 PMC6800799

[30] Barrenetxea J, Tan KB, Tong R, Chua K, Feng Q, Koh W-P, et al. Emergency hospital admissions among older adults living alone in the community. BMC Health Serv Res. 2021;21(1):1192. doi: 10.1186/s12913-021-07216-3 34732180 PMC8567640

[31] Dreyer K, Steventon A, Fisher R, Deeny SR. The association between living alone and health care utilisation in older adults: a retrospective cohort study of electronic health records from a London general practice. BMC Geriatr. 2018;18(1):269. doi: 10.1186/s12877-018-0939-4 30514225 PMC6280341

[32] Pandey KR, Yang F, Cagney KA, Smieliauskas F, Meltzer DO, Ruhnke GW. The impact of marital status on health care utilization among Medicare beneficiaries. Medicine (Baltimore). 2019;98(12):e14871. doi: 10.1097/MD.0000000000014871 30896632 PMC6709281

[33] Gerst-Emerson K, Jayawardhana J. Loneliness as a public health issue: the impact of loneliness on health care utilization among older adults. Am J Public Health. 2015;105(5):1013–9. doi: 10.2105/AJPH.2014.302427 25790413 PMC4386514

[34] Valtorta NK, Moore DC, Barron L, Stow D, Hanratty B. Older Adults’ Social Relationships and Health Care Utilization: A Systematic Review. Am J Public Health. 2018;108(4):e1–10. doi: 10.2105/AJPH.2017.304256 29470115 PMC5844393

[35] Gao J, Moran E, Grimm R, Toporek A, Ruser C. The Effect of Primary Care Visits on Total Patient Care Cost: Evidence From the Veterans Health Administration. J Prim Care Community Health. 2022;13:21501319221141792. doi: 10.1177/21501319221141792 36564889 PMC9793026

[36] Gavulic KA, Gonzales G. Health Care Expenditures and Financial Burden: A Comparison of Adults in Same-Sex Couples and Different-Sex Couples. Med Care Res Rev. 2022;79(2):281–9. doi: 10.1177/10775587211004308 33783242

[37] Blosnich JR, Farmer GW, Lee JGL, Silenzio VMB, Bowen DJ. Health inequalities among sexual minority adults evidence from ten US states, 2010. Am J Prev Med. 2014;46:337–49. 24650836 10.1016/j.amepre.2013.11.010 PMC4102129

[38] Frimpong EY, Rowan GA, Williams D, Li M, Solano L, Chaudhry S, et al. Health Disparities, Inpatient Stays, and Emergency Room Visits Among Lesbian, Gay, and Bisexual People: Evidence From a Mental Health System. Psychiatr Serv. 2020;71(2):128–35. doi: 10.1176/appi.ps.201900188 31590623

[39] Gonzales G, Henning-Smith C. Health Disparities by Sexual Orientation: Results and Implications from the Behavioral Risk Factor Surveillance System. J Community Health. 2017;42(6):1163–72. doi: 10.1007/s10900-017-0366-z 28466199

[40] Short ME, Goetzel RZ, Pei X, Tabrizi MJ, Ozminkowski RJ, Gibson TB, et al. How accurate are self-reports? Analysis of self-reported health care utilization and absence when compared with administrative data. J Occup Environ Med. 2009;51(7):786–96. doi: 10.1097/JOM.0b013e3181a86671 19528832 PMC2745402

[41] Muggah E, Graves E, Bennett C, Manuel DG. Ascertainment of chronic diseases using population health data: a comparison of health administrative data and patient self-report. BMC Public Health. 2013;13:16. doi: 10.1186/1471-2458-13-16 23302258 PMC3557162

[42] Grimwood CL, Holland AE, McDonald CF, Mahal A, Hill CJ, Lee AL, et al. Comparison of self-report and administrative data sources to capture health care resource use in people with chronic obstructive pulmonary disease following pulmonary rehabilitation. BMC Health Serv Res. 2020;20(1):1061. doi: 10.1186/s12913-020-05920-0 33228654 PMC7682690

[43] Virnig B. Strengths and limitations of CMS administrative data in research. ResDAC; 2018.

[44] Card DE, Chetty R, Feldstein MS, Saez E. Expanding Access to Administrative Data for Research in the United States. SSRN Journal. 2010. doi: 10.2139/ssrn.1888586

[45] Mazzali C, Duca P. Use of administrative data in healthcare research. Intern Emerg Med. 2015;10(4):517–24. doi: 10.1007/s11739-015-1213-9 25711312

[46] Dragon CN, Guerino P, Ewald E, Laffan AM. Transgender Medicare Beneficiaries and Chronic Conditions: Exploring Fee-for-Service Claims Data. LGBT Health. 2017;4(6):404–11. doi: 10.1089/lgbt.2016.0208 29125908 PMC5731542

[47] Progovac AM, Cook BL, Mullin BO, McDowell A, Sanchez R MJ, Wang Y, et al. Identifying Gender Minority Patients’ Health And Health Care Needs In Administrative Claims Data. Health Aff (Millwood). 2018;37(3):413–20. doi: 10.1377/hlthaff.2017.1295 29505378 PMC5942884

[48] Progovac AM, Mullin BO, Creedon TB, McDowell A, Sanchez-Roman MJ, Hatfield LA, et al. Trends in Mental Health Care Use in Medicare from 2009 to 2014 by Gender Minority and Disability Status. LGBT Health. 2019;6(6):297–305. doi: 10.1089/lgbt.2018.0221 31436481 PMC6740156

[49] Fredriksen-Goldsen KI, Simoni JM, Kim H-J, Lehavot K, Walters KL, Yang J, et al. The health equity promotion model: Reconceptualization of lesbian, gay, bisexual, and transgender (LGBT) health disparities. Am J Orthopsychiatry. 2014;84(6):653–63. doi: 10.1037/ort0000030 25545433 PMC4350932

[50] Joynt KE, Figueroa JF, Beaulieu N, Wild RC, Orav EJ, Jha AK. Segmenting high-cost Medicare patients into potentially actionable cohorts. Healthc (Amst). 2017;5(1–2):62–7. doi: 10.1016/j.hjdsi.2016.11.002 27914968

[51] Rivera-Hernandez M, Kumar A, Chou L-N, Keeney T, Ferdows N, Karmarkar A, et al. Healthcare utilization and costs among high-need and frail Mexican American Medicare beneficiaries. PLoS One. 2022;17(1):e0262079. doi: 10.1371/journal.pone.0262079 35030180 PMC8759642

[52] Casalino LP, Ramsay P, Baker LC, Pesko MF, Shortell SM. Medical Group Characteristics and the Cost and Quality of Care for Medicare Beneficiaries. Health Serv Res. 2018;53(6):4970–96. doi: 10.1111/1475-6773.13010 29978481 PMC6232442

[53] Figueroa JF, Zhou X, Jha AK. Characteristics And Spending Patterns Of Persistently High-Cost Medicare Patients. Health Aff (Millwood). 2019;38(1):107–14. doi: 10.1377/hlthaff.2018.05160 30615516

[54] Hughes ME, Waite LJ, Hawkley LC, Cacioppo JT. A Short Scale for Measuring Loneliness in Large Surveys: Results From Two Population-Based Studies. Res Aging. 2004;26(6):655–72. doi: 10.1177/0164027504268574 18504506 PMC2394670

[55] Lachman ME, Weaver SL. The sense of control as a moderator of social class differences in health and well-being. J Pers Soc Psychol. 1998;74(3):763–73. doi: 10.1037//0022-3514.74.3.763 9523418

[56] Kim H-J, Romanelli M, Fredriksen-Goldsen K. Multidimensional social connectedness of sexual and gender minority midlife and older adults: Findings from the National Health, Aging, and Sexuality/Gender Study (NHAS). Am J Orthopsychiatry. 2024;94(3):322–38. doi: 10.1037/ort0000726 38300585 PMC11149496

[57] Sherbourne CD, Stewart AL. The MOS social support survey. Soc Sci Med. 1991;32(6):705–14. doi: 10.1016/0277-9536(91)90150-b 2035047

[58] U. S. Department of Health and Human Services. U.S Department of Health and Human Services Implementation Guidance on Data Collection Standards for Race, Ethnicity, Sex, Primary Language, and Disability Status. 2011.

[59] CDC. Vital Signs: Current Cigarette Smoking Among Adults Aged ≥18 Years --- United States, 2005--2010 2011. [accessed November 26, 2024]. https://www.cdc.gov/mmwr/preview/mmwrhtml/mm6035a5.htm

[60] U. S. Department of Health and Human Services. Annual update of the HHS poverty guidelines. Federal Register. 2013;78:5182–3.

[61] Figueroa JF, Katz IT, Hyle EP, Horneffer KE, Nambiar K, Phelan J, et al. The Association Of HIV With Health Care Spending And Use Among Medicare Beneficiaries. Health Aff (Millwood). 2022;41(4):581–8. doi: 10.1377/hlthaff.2021.01793 35377765 PMC9153068

[62] Figueroa JF, Phelan J, Orav EJ, Patel V, Jha AK. Association of Mental Health Disorders With Health Care Spending in the Medicare Population. JAMA Netw Open. 2020;3(3):e201210. doi: 10.1001/jamanetworkopen.2020.1210 32191329 PMC7082719

[63] Malehi AS, Pourmotahari F, Angali KA. Statistical models for the analysis of skewed healthcare cost data: a simulation study. Health Econ Rev. 2015;5:11. doi: 10.1186/s13561-015-0045-7 26029491 PMC4442782

[64] Bradley EH, Canavan M, Rogan E, Talbert-Slagle K, Ndumele C, Taylor L, et al. Variation In Health Outcomes: The Role Of Spending On Social Services, Public Health, And Health Care, 2000-09. Health Aff (Millwood). 2016;35(5):760–8. doi: 10.1377/hlthaff.2015.0814 27140980

[65] Cubanski J, Freed M, Ochieng N, Cottrill A, Fuglesten Biniek J, Neuman T. How much does Medicare spend and how is the program financed? - Medicare 101. 2024.

[66] Cubanski J, Neuman T. The fact on Medicare spending and financing. Kaiser Family Foundation; 2017.

[67] Sambamoorthi U, Tan X, Deb A. Multiple chronic conditions and healthcare costs among adults. Expert Rev Pharmacoecon Outcomes Res. 2015;15(5):823–32. doi: 10.1586/14737167.2015.1091730 26400220 PMC4698815

[68] Buttorff C, Ruder T, Bauman M. Multiple chronic conditions in the United States. 2017.

[69] Lehnert T, Heider D, Leicht H, Heinrich S, Corrieri S, Luppa M, et al. Review: health care utilization and costs of elderly persons with multiple chronic conditions. Med Care Res Rev. 2011;68(4):387–420. doi: 10.1177/1077558711399580 21813576

[70] Barrio-Cortes J, Castaño-Reguillo A, Beca-Martínez MT, Bandeira-de Oliveira M, López-Rodríguez C, Jaime-Sisó MÁ. Chronic diseases in the geriatric population: morbidity and use of primary care services according to risk level. BMC Geriatr. 2021;21(1):278. doi: 10.1186/s12877-021-02217-7 33902470 PMC8074273

[71] Kastner M, Cardoso R, Lai Y, Treister V, Hamid JS, Hayden L, et al. Effectiveness of interventions for managing multiple high-burden chronic diseases in older adults: a systematic review and meta-analysis. CMAJ. 2018;190(34):E1004–12. doi: 10.1503/cmaj.171391 30150242 PMC6110649

[72] MedPAC, MACPAC. Data book: Beneficiaries dually eligible for Medicare and Medicaid. 2024.

[73] Fredriksen-Goldsen KI, Kim H-J, Barkan SE. Disability among lesbian, gay, and bisexual adults: disparities in prevalence and risk. Am J Public Health. 2012;102(1):e16-21. doi: 10.2105/AJPH.2011.300379 22095356 PMC3490559

[74] Peikes DN, Swankoski KE, Rastegar JS, Franklin SM, Pavliv DJ. Burden Of Health-Related Social Needs Among Dual- And Non-Dual-Eligible Medicare Advantage Beneficiaries. Health Aff (Millwood). 2023;42(7):899–908. doi: 10.1377/hlthaff.2022.01574 37406240

[75] Lampe NM, Barbee H, Tran NM, Bastow S, McKay T. Health Disparities Among Lesbian, Gay, Bisexual, Transgender, and Queer Older Adults: A Structural Competency Approach. Int J Aging Hum Dev. 2024;98(1):39–55. doi: 10.1177/00914150231171838 37122150 PMC10598237

[76] Srithumsuk W, Thummapol O, Bhatarasakoon P. Social Determinants of Health Inequities for Older LGBT Adults: A Scoping Review. J Transcult Nurs. 2024;35(5):368–80. doi: 10.1177/10436596241253866 38767232

[77] Choi NG, Marti CNN, DiNitto DM, Choi BY. Alcohol Use as Risk Factors for Older Adults’ Emergency Department Visits: A Latent Class Analysis. West J Emerg Med. 2015;16(7):1146–58. doi: 10.5811/westjem.2015.9.27704 26759670 PMC4703192

[78] Gellert C, Schöttker B, Brenner H. Smoking and all-cause mortality in older people: systematic review and meta-analysis. Arch Intern Med. 2012;172(11):837–44. doi: 10.1001/archinternmed.2012.1397 22688992

[79] Esser MB, Sherk A, Liu Y, Naimi TS. Deaths from Excessive Alcohol Use - United States, 2016-2021. MMWR Morb Mortal Wkly Rep. 2024;73(8):154–61. doi: 10.15585/mmwr.mm7308a1 38421934 PMC10907037

[80] Hubbard RE, Searle SD, Mitnitski A, Rockwood K. Effect of smoking on the accumulation of deficits, frailty and survival in older adults: a secondary analysis from the Canadian Study of Health and Aging. J Nutr Health Aging. 2009;13(5):468–72. doi: 10.1007/s12603-009-0085-y 19390755 PMC12880272

[81] Erdley SD, Anklam DD, Reardon CC. Breaking barriers and building bridges: understanding the pervasive needs of older LGBT adults and the value of social work in health care. J Gerontol Soc Work. 2014;57(2–4):362–85. doi: 10.1080/01634372.2013.871381 24329570

[82] Jackson SE, Hackett RA, Grabovac I, Smith L, Steptoe A. Perceived discrimination, health and wellbeing among middle-aged and older lesbian, gay and bisexual people: A prospective study. PLoS One. 2019;14(5):e0216497. doi: 10.1371/journal.pone.0216497 31075153 PMC6510440

[83] Fredriksen-Goldsen KI, Hoy-Ellis CP, Goldsen J, Emlet CA, Hooyman NR. Creating a vision for the future: key competencies and strategies for culturally competent practice with lesbian, gay, bisexual, and transgender (LGBT) older adults in the health and human services. J Gerontol Soc Work. 2014;57(2–4):80–107. doi: 10.1080/01634372.2014.890690 24571387 PMC4091982

[84] Fredriksen-Goldsen KI, Shiu C, Bryan AEB, Goldsen J, Kim H-J. Health Equity and Aging of Bisexual Older Adults: Pathways of Risk and Resilience. J Gerontol B Psychol Sci Soc Sci. 2017;72(3):468–78. doi: 10.1093/geronb/gbw120 27815302 PMC5927101

[85] MAP & SAGE. Understanding issues facing LGBT older adults 2017. https://www.lgbtmap.org/policy-and-issue-analysis/understanding-issues-facing-lgbt-older-adults

[86] Phan M, Graham C, Moffett T, Flatt J. Meeting the health and social needs of LGBTQ older adults through Medicaid. Center for Health Care Strategies; 2023.

[87] Chen J, McLaren H, Jones M, Shams L. The Aging Experiences of LGBTQ Ethnic Minority Older Adults: A Systematic Review. Gerontologist. 2022;62(3):e162–77. doi: 10.1093/geront/gnaa134 32941597

[88] Starfield B, Shi L, Macinko J. Contribution of primary care to health systems and health. Milbank Q. 2005;83(3):457–502. doi: 10.1111/j.1468-0009.2005.00409.x 16202000 PMC2690145

[89] Sawicki OA, Mueller A, Klaaßen-Mielke R, Glushan A, Gerlach FM, Beyer M, et al. Strong and sustainable primary healthcare is associated with a lower risk of hospitalization in high risk patients. Sci Rep. 2021;11(1):4349. doi: 10.1038/s41598-021-83962-y 33623130 PMC7902818

[90] Reschovsky JD, Hadley J, Saiontz-Martinez CB, Boukus ER. Following the money: factors associated with the cost of treating high-cost Medicare beneficiaries. Health Serv Res. 2011;46(4):997–1021. doi: 10.1111/j.1475-6773.2011.01242.x 21306368 PMC3165175

[91] Khullar D, Zhang Y, Kaushal R. Potentially Preventable Spending Among High-Cost Medicare Patients: Implications for Healthcare Delivery. J Gen Intern Med. 2020;35(10):2845–52. doi: 10.1007/s11606-020-05691-8 32103440 PMC7573047

[92] CDC. HIV Surveillance Report: Diagnoses of HIV Infection in the United States and Dependent Areas, 2021. 2021.

[93] Cylus J, Hartman M, Washington B, Andrews K, Catlin A. Pronounced gender and age differences are evident in personal health care spending per person. Health Aff (Millwood). 2011;30(1):153–60. doi: 10.1377/hlthaff.2010.0216 21148180

[94] Lassman D, Hartman M, Washington B, Andrews K, Catlin A. US health spending trends by age and gender: selected years 2002-10. Health Aff (Millwood). 2014;33(5):815–22. doi: 10.1377/hlthaff.2013.1224 24799579

[95] Shugarman LR, Bird CE, Schuster CR, Lynn J. Age and gender differences in medicare expenditures and service utilization at the end of life for lung cancer decedents. Womens Health Issues. 2008;18(3):199–209. doi: 10.1016/j.whi.2008.02.008 18457755 PMC2440649

[96] Emlet CA. “You’re awfully old to have this disease”: experiences of stigma and ageism in adults 50 years and older living with HIV/AIDS. Gerontologist. 2006;46(6):781–90. doi: 10.1093/geront/46.6.781 17169933

[97] Fredriksen-Goldsen KI. Resilience and Disparities among Lesbian, Gay, Bisexual, and Transgender Older Adults. Public Policy Aging Rep. 2011;21(3):3–7. doi: 10.1093/ppar/21.3.3 26755892 PMC4706747

