# Supplementary material for: Chronic conditions and healthcare cost and utilization among underserved Medicare beneficiaries
Source: PLoS One. 2026 Feb 26;21(2):e0340785. doi: 10.1371/journal.pone.0340785 (PMC12944782; doi:10.1371/journal.pone.0340785)
Supplement: S1 Table — (DOCX) [file pone.0340785.s001.docx]

**S1 Table. CMS Cost and Use service claims used in Medicare spending and utilization**

| **Type of Claim in CMS Cost and Use File** |
| --- |
| Acute Inpatient |
| Other Inpatient |
| Hospital Outpatient |
| Part B Physician Services* |
| Evaluation and Management* |
| Home Health |
| Skilled Nursing Facility |
| Ambulatory Surgery Center |
| Hospice |
| Dialysis |
| Other Procedures |
| Imaging |
| Tests |
| Durable Medical Equipment |
| Anesthesia |
| Part B Drug Beneficiary |
| Part D |

*Note:* Part B Physician Services and Evaluation and Management were used to calculate spending on physician services per beneficiary
