# Supplementary material for: Chronic conditions and healthcare cost and utilization among underserved Medicare beneficiaries
Source: PLoS One. 2026 Feb 26;21(2):e0340785. doi: 10.1371/journal.pone.0340785 (PMC12944782; doi:10.1371/journal.pone.0340785)
Supplement: S2 Table — (DOCX) [file pone.0340785.s002.docx]

**S2 Table. Classification of Chronic Conditions based on CMS Chronic Conditions and Other Chronic Conditions files**

| **Complex Chronic Conditions** | **Other Chronic Conditions** |
| --- | --- |
| Acute Myocardial Infarction | Hip/Pelvic Fracture |
| Ischemic Heart Disease | Mobility Impairments |
| Chronic Kidney Disease | Rheumatoid Arthritis/ Osteoarthritis |
| Congestive Heart Failure | Benign Prostatic Hyperplasia |
| Alzheimer’s Disease, Related Disorders, or Senile Dementia | Colorectal, Endometrial, Breast, Lung, and Prostate Cancer |
| Diabetes | Leukemia and Lymphoma |
| Chronic Obstructive Pulmonary Disease | Obesity |
| Schizophrenia | Cataracts |
| Depression | Glaucoma |
| Bipolar Disorder | Anemia |
| Personality Disorders | Hyperlipidemia |
| Atrial Fibrillation | Hypertension |
| Stroke/ Transient Ischemic Attack | Liver Disease, Cirrhosis, and Other Liver Conditions |
|  | Multiple Sclerosis |
|  | Muscular Dystrophy |
|  | Osteoporosis |
|  | Pressure and Chronic Ulcers |
|  | Acquired Hypothyroidism |
|  | Cerebral Palsy |

*Note 1: Rivera-Hernandez et al. (2022) also included Cystic Fibrosis and Drug Use Disorders as part of Other Chronic Conditions diagnoses. Acquired data files from CMS did not include these diagnosis categories and so are not included.*

*Note 2: No participants in the sample were ever diagnosed with muscular dystrophy or cerebral palsy.*
