# Supplementary material for: Chronic conditions and healthcare cost and utilization among underserved Medicare beneficiaries
Source: PLoS One. 2026 Feb 26;21(2):e0340785. doi: 10.1371/journal.pone.0340785 (PMC12944782; doi:10.1371/journal.pone.0340785)
Supplement: S3 Table — (DOCX) [file pone.0340785.s003.docx]

**S3 Table. Bivariate linear regression of log-transformed total Medicare spending and total physician spending on chronic conditions (N = 902; exponentiated coefficients)**

|  | Total Medicare Spending | | | Total Spending on Physician Services | | |
| --- | --- | --- | --- | --- | --- | --- |
|  | Exp(β) | 95% CI | *p* | Exp(β) | 95% CI | *p* |
| Chronic Conditions Group |  |  |  |  |  |  |
| Major Complex Chronic Illness | 412.71 | (211.84, 80.04) | <.001 | 23,769.85 | (11,440.49, 49,386.48) | <.001 |
| Minor Complex Chronic Illness | 80.35 | (41.27, 156.44) | <.001 | 1,599.70 | (770.52, 3,321.20) | <.001 |
| Simple Chronic Illness | 37.96 | (16.94, 85.09) | <.001 | 602.83 | (248.79, 1,460.63) | <.001 |

Note. CI = confidence interval
