# Supplementary material for: Chronic conditions and healthcare cost and utilization among underserved Medicare beneficiaries
Source: PLoS One. 2026 Feb 26;21(2):e0340785. doi: 10.1371/journal.pone.0340785 (PMC12944782; doi:10.1371/journal.pone.0340785)
Supplement: S4 Table — (DOCX) [file pone.0340785.s004.docx]

**S4 Table. Linear regressions of log-transformed healthcare spending on chronic conditions and background characteristics/social location**

|  | Total Medicare Spending | | | Total Spending on Physician Services | | |
| --- | --- | --- | --- | --- | --- | --- |
|  | Exp(β) | 95% CI | *p* | Exp(β) | 95% CI | *p* |
| Chronic Conditions Group |  |  |  |  |  |  |
| Major Complex Chronic Illness | 424.27 | (217.14, 828.97) | <.001 | 25,865.20 | (12,429.05, 53,826.17) | <.001 |
| Minor Complex Chronic Illness | 81.68 | (41.77, 159.74) | <.001 | 1,620.74 | (778.05, 3,376.14) | <.001 |
| Simple Chronic Illness | 40.53 | (17.99, 91.34) | <.001 | 578.72 | (237.92, 1,407.68) | <.001 |
| Comparatively Healthy | Referent |  |  | Referent |  |  |
| Background Characteristics/Social Location |  |  |  |  |  |  |
| Age | 0.97 | (0.93, 1.00) | .061 | 0.96 | (0.92, 1.00) | .054 |
| Gender |  |  |  |  |  |  |
| Women | Referent |  |  | Referent |  |  |
| Men | 1.16 | (0.68, 1.98) | .578 | 0.64 | (0.36, 1.15) | .136 |
| Gender Diverse | 2.89 | (0.39, 21.62) | .302 | 1.46 | (0.16, 13.17) | .739 |
| Sexual Identity |  |  |  |  |  |  |
| Lesbian/gay | Referent |  |  | Referent |  |  |
| Bisexual | 0.75 | (0.28, 1.98) | .556 | 0.58 | (0.20, 1.69) | .317 |
| Sexual Diverse | 0.55 | (0.11, 2.64) | .451 | 0.46 | (0.08, 2.58) | .377 |
| Gender Identity |  |  |  |  |  |  |
| Transgender | 084 | (0.20, 3.46) | .806 | 2.05 | (0.43, 9.67) | .367 |
| Race/Ethnicity |  |  |  |  |  |  |
| Non-Hispanic White | Referent |  |  | Referent |  |  |
| Hispanic | 2.17 | (0.67, 7.02) | .195 | 0.97 | (0.27, 3.49) | .959 |
| Black or African American | 0.37 | (0.12, 1.11) | .077 | 0.30 | (0.09, 0.98) | .047 |
| Other | 1.97 | (0.61, 6.31) | .254 | 0.71 | (0.20, 2.54) | .599 |

Note. CI = confidence interval
