# Supplementary material for: Chronic conditions and healthcare cost and utilization among underserved Medicare beneficiaries
Source: PLoS One. 2026 Feb 26;21(2):e0340785. doi: 10.1371/journal.pone.0340785 (PMC12944782; doi:10.1371/journal.pone.0340785)
Supplement: S5 Table — (DOCX) [file pone.0340785.s005.docx]

**S5 Table. Linear regressions of log-transformed healthcare spending on chronic conditions, background characteristics/social location, adverse experiences, psychological and social resources, health-related indicators, and socioeconomic factors.**

|  | Total Medicare Spending | | | Total Spending on Physician Services | | |
| --- | --- | --- | --- | --- | --- | --- |
|  | Exp(β) | 95% CI | *p* | Exp(β) | 95% CI | *p* |
| Chronic Conditions Group |  |  |  |  |  |  |
| Major Complex Chronic Illness | 204.16 | (98.52, 423.08) | <.001 | 44,347.00 | (19,803.41, 99,308.96) | <.001 |
| Minor Complex Chronic Illness | 50.03 | (29.12, 115.66) | <.001 | 2,140.31 | (997.93, 4,590.45) | <.001 |
| Simple Chronic Illness | 45.74 | (20.22, 103.49) | <.001 | 584.38 | (236.78, 1,442.24) | <.001 |
| Background Characteristics/ Social Location |  |  |  |  |  |  |
| Age | 0.99 | (0.94, 1.04) | .637 | 0.89 | (0.84, 0.95) | <.001 |
| Gender |  |  |  |  |  |  |
| Men | 1.17 | (0.65, 2.12) | .603 | 0.61 | (0.32, 1.18) | .143 |
| Gender Diverse | 3.92 | (0.52, 29.80) | .187 | 0.53 | (0.06, 4.97) | .576 |
| Sexual Identity |  |  |  |  |  |  |
| Bisexual | 0.86 | (0.31, 2.36) | .767 | 0.70 | (0.23, 2.14) | .534 |
| Sexual Diverse | 0.23 | (0.04, 1.21) | .083 | 0.38 | (0.06, 2.36) | .297 |
| Gender Identity |  |  |  |  |  |  |
| Transgender | 0.72 | (0.17, 3.09) | .657 | 4.36 | (0.87, 21.91) | .074 |
| Race/Ethnicity |  |  |  |  |  |  |
| Hispanic | 1.29 | (0.37, 4.45) | .690 | 1.28 | (0.33, 5.07) | .720 |
| Black or African American | 0.20 | (0.06, 0.65) | .007 | 0.26 | (0.07, 0.96) | .043 |
| Other | 0.91 | (0.28, 3.02) | .879 | 0.96 | (0.26, 3.62) | .955 |
| Adverse Experiences |  |  |  |  |  |  |
| Lifetime Victimization | 1.01 | (0.95, 1.08) | .744 | 1.00 | (0.93, 1.07) | .954 |
| Lifetime Discrimination | 0.94 | (0.82, 1.07) | .328 | 0.99 | (0.86, 1.15) | .928 |
| Day-to-day Discrimination | 0.82 | (0.53, 1.27) | .372 | 1.55 | (0.96, 2.51) | .073 |
| Stigma | 0.92 | (0.64, 1.34) | .676 | 1.06 | (0.70, 1.61) | .770 |
| Loneliness | 0.97 | (0.68, 1.38) | .868 | 0.65 | (0.44, 0.96) | .029 |
| Psychological and Social Resources |  |  |  |  |  |  |
| Mastery | 1.04 | (0.75, 1.45) | .819 | 0.85 | (0.59, 1.23) | .392 |
| Marital/Partner Status - Partnered | 0.99 | (0.47, 2.12) | .988 | 0.86 | (0.37, 1.99) | .730 |
| Living alone - Yes | 1.08 | (0.54, 2.17) | .823 | 0.88 | (0.41, 1.90) | .747 |
| Social network size | 0.97 | (0.92, 1.02) | .269 | 1.02 | (0.96, 1.07) | .604 |
| Social support | 0.76 | (0.53, 1.09) | .130 | 0.89 | (0.60, 1.33) | .567 |
| Community Engagement | 1.27 | (1.00, 1.62) | .055 | 0.88 | (0.68, 1.16) | .369 |
| Health-Related Indicators |  |  |  |  |  |  |
| Disability - Yes | 1.87 | (1.02, 3.43) | .043 | 0.96 | (0.49, 1.88) | .910 |
| Physical Impairment | 1.47 | (0.97, 1.01) | .071 | 0.69 | (0.43, 1.09) | .109 |
| Cognitive Impairment | 0.99 | (0.97, 1.01) | .453 | 1.00 | (0.98, 1.03) | .676 |
| Smoking - Yes | 0.85 | (0.25, 2.83) | .787 | 0.51 | (0.13, 1.92) | .317 |
| Binge Drinking - Yes | 0.35 | (0.16, 0.76) | .008 | 0.67 | (0.28, 1.58) | .360 |
| Current Reason for Medicare Entitlement – Disability | 2.35 | (0.81, 6.84) | .118 | 0.22 | (0.07, 0.73) | .013 |
| Socioeconomic Factors |  |  |  |  |  |  |
| Income, =< 200% FPG | 0.52 | (0.27, 1.01) | .054 | 0.22 | (0.11, 0.46) | <.001 |
| Dually Eligible for Medicaid -Enrolled | 5.12 | (2.08, 1.01) | <.001 | 1.91 | (0.71, 5.17) | .203 |
| Currently Employed | 0.19 | (2.08, 12.61) | <.001 | 0.89 | (0.45, 1.79) | .752 |
| Education |  |  |  |  |  |  |
| Some College | 1.63 | (0.56, 4.72) | .367 | 2.43 | (0.75, 7.88) | .139 |
| Graduate or Professional Degree | 2.28 | (0.75, 6.92) | .146 | 2.69 | (0.79, 9.19) | .114 |

Note. CI = confidence interval; FPG = federal poverty guideline
